# Exploring the Vibrational Side of Spin‐Phonon Coupling in Single‐Molecule Magnets via ^161^Dy Nuclear Resonance Vibrational Spectroscopy

**DOI:** 10.1002/anie.201914728

**Published:** 2020-04-24

**Authors:** Lena Scherthan, Rouven F. Pfleger, Hendrik Auerbach, Tim Hochdörffer, Juliusz A. Wolny, Wenli Bi, Jiyong Zhao, Michael Y. Hu, E. Ercan Alp, Christopher E. Anson, Rolf Diller, Annie K. Powell, Volker Schünemann

**Affiliations:** ^1^ Department of Physics Technische Universität Kaiserslautern Erwin-Schrödinger-Str. 46 67663 Kaiserslautern Germany; ^2^ Institute of Inorganic Chemistry Karlsruhe Institute of Technology Engesserstr. 15 76131 Karlsruhe Germany; ^3^ Advanced Photon Source Argonne National Laboratory 9700 South Cass Avenue Argonne IL 60439 USA; ^4^ Institute of Nanotechnology Karlsruhe Institute of Technology 76021 Karlsruhe Germany; ^5^ Department of Physics University of Alabama at Birmingham Birmingham AL 35294 USA

**Keywords:** dysprosium, nuclear resonance vibrational spectroscopy, phonons, single-molecule magnets

## Abstract

Synchrotron‐based nuclear resonance vibrational spectroscopy (NRVS) using the Mössbauer isotope ^161^Dy has been employed for the first time to study the vibrational properties of a single‐molecule magnet (SMM) incorporating Dy^III^, namely [Dy(Cy_3_PO)_2_(H_2_O)_5_]Br_3_⋅2 (Cy_3_PO)⋅2 H_2_O ⋅2 EtOH. The experimental partial phonon density of states (pDOS), which includes all vibrational modes involving a displacement of the Dy^III^ ion, was reproduced by means of simulations using density functional theory (DFT), enabling the assignment of all intramolecular vibrational modes. This study proves that ^161^Dy NRVS is a powerful experimental tool with significant potential to help to clarify the role of phonons in SMMs.

## Graphical abstract

**Bad vibrations?** ^161^Dy nuclear resonance vibrational spectroscopy gives direct experimental access to the partial phonon density of states which includes all vibrational modes involving a displacement of the Dy^III^ ion. In combination with density functional theory, an assignment to all intramolecular vibrational modes is possible, paving an ideal path to help to clarify the role of phonons in single‐molecule magnets.

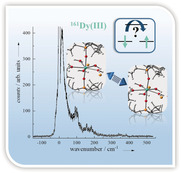

In the emerging field of quantum technologies aiming for potential applications in data storage and quantum computing, molecular magnetic materials such as spin qubits, spin‐crossover compounds, and single‐molecule magnets (SMMs) arouse a great deal of interest.^1^, ^2^, ^3^, ^4^ Until recently, the main focus of experimental and theoretical studies on SMMs was placed on the spin number and height of the energy barrier to spin reversal.^1^, ^2^, ^4^, ^5^, ^6^ Now, it has been pointed out that the interaction of the electronic spins with their environment needs to be taken into account since the interplay of spin relaxation processes with vibrational modes and lattice phonons offers pathways for quantum tunneling of the magnetization (QTM) such that the system can completely (ground‐state QTM) or partially (thermally assisted QTM) avoid going over the energy barrier to spin reversal.^1^, ^5^, ^6^, ^7^, ^8^ Thus, spin‐phonon coupling, accounting for modulations of the ligand field owing to ligand displacements relative to the metal center, is of immense importance.^6^, ^9^ A recent perspective article summarizing the state of the art and suggesting ways forward points out the importance of this coupling as a key parameter for the description of relaxation processes, especially at higher temperatures.^8^ Moreover, the importance of intramolecular vibrations with respect to spin dynamics was underlined in multiple independent theoretical studies.^5^, ^10^ Besides the theoretical models, some experimental investigations also indicate that intramolecular vibrational modes are involved in the relaxation process in SMMs.^11^, ^12^, ^13^

Here we describe how the use of the synchrotron based technique nuclear resonance vibrational spectroscopy (NRVS) contributes to this research by providing insight about the phonon density of states (DOS) which is key to unraveling relaxation processes modulated by intermolecular acoustic and intramolecular optical phonons (molecular vibrational modes).^14^

NRVS, also referred to as nuclear inelastic scattering (NIS) or nuclear resonant inelastic X‐ray scattering (NRIXS), uses a Mössbauer active nucleus as a local probe in order to detect vibrational properties of a material.^16^ More precisely, all modes that include a Dy displacement are considered, which is why the resulting DOS is also referred to as partial DOS (pDOS).^28^ In contrast to other well‐established methods such as IR and Raman spectroscopy, NRVS has only one less limiting selection rule.^17^ This rule, namely the requirement of a non‐zero projection of the movement of the resonant Mössbauer nuclei onto the direction of the incident synchrotron beam, is fortunately of minor importance for powder samples.^18^ This makes NRVS an ideal means for observing the contribution of vibrational/phonon modes to the energy landscape of a system, since NRVS is a means to directly detect the energy transfer for a system by exciting the relevant Mössbauer nucleus and then using the inelastic energy changes to explore the vibrational and phonon modes^17^, ^19^ which are known to mediate magnetic relaxation pathways.^8^ Superior to neutron scattering, NRVS has been shown to have monolayer sensitivity^20^ which in principle enables investigation of metal complexes in interaction with surfaces.

Given the intense interest in using dysprosium in the fields of solid‐state^21^ and thin‐film magnetism,^22^ but especially in the research field of SMMs,^3^, ^9^, ^23^ we have used ^161^Dy NRVS for the first time for the detection of phonons of a Dy^III^ coordination complex which shows slow relaxation of its magnetization. We have thus selected a suitable prototype‐like single‐ion magnet, namely [Dy(Cy_3_PO)_2_(H_2_O)_5_]Br_3_⋅2 (Cy_3_PO)⋅2 H_2_O⋅2 EtOH^15^, ^24^ (Cy_3_PO=tricyclohexyl phosphine oxide) (**1**), to establish ^161^Dy NRVS in SMM research. This compound has a pentagonal‐bipyramidal geometry which leads to favorable axiality (see Figure 1). It exhibits well‐defined SMM behavior with magnetic blocking up to a temperature of 20 K in zero dc field, which means that there is no appreciable zero‐field QTM.^15^

**Figure 1:**
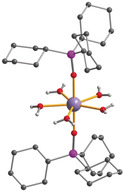
Molecular structure of complex **1** (redrawn from coordinates in ref. 15 cyclohexyl H atoms omitted for clarity). Dy: purple, O: red, P: pink, C: gray, H: white.

The results presented here were obtained by performing experiments at the beamline 3‐ID‐B at the Advanced Photon Source (APS) at Argonne National Laboratory (see the Experimental Section in the Supporting Information). In order to achieve an adequate signal, the polycrystalline sample **1** was synthesized using 91 % enriched ^161^Dy_2_O_3_. Details of the synthetic procedure are given in the Supporting Information.

Figure 2 shows the ^161^Dy NRVS data of **1** recorded at *T=*21(3) K (for temperature determination see the Supporting Information). The phonon creation part of this spectrum exhibits a sharp low‐energy peak at 16 cm^−1^, with several maxima of intensity on its shoulder (see inset in Figure 2). Furthermore, a broadened mode at around 100 cm^−1^ with three maxima separated by less than 7–8 cm^−1^ is observed. Several further maxima of intensity exist up to 200 cm^−1^, followed by three more peaks between 300 cm^−1^ and 400 cm^−1^.

**Figure 2:**
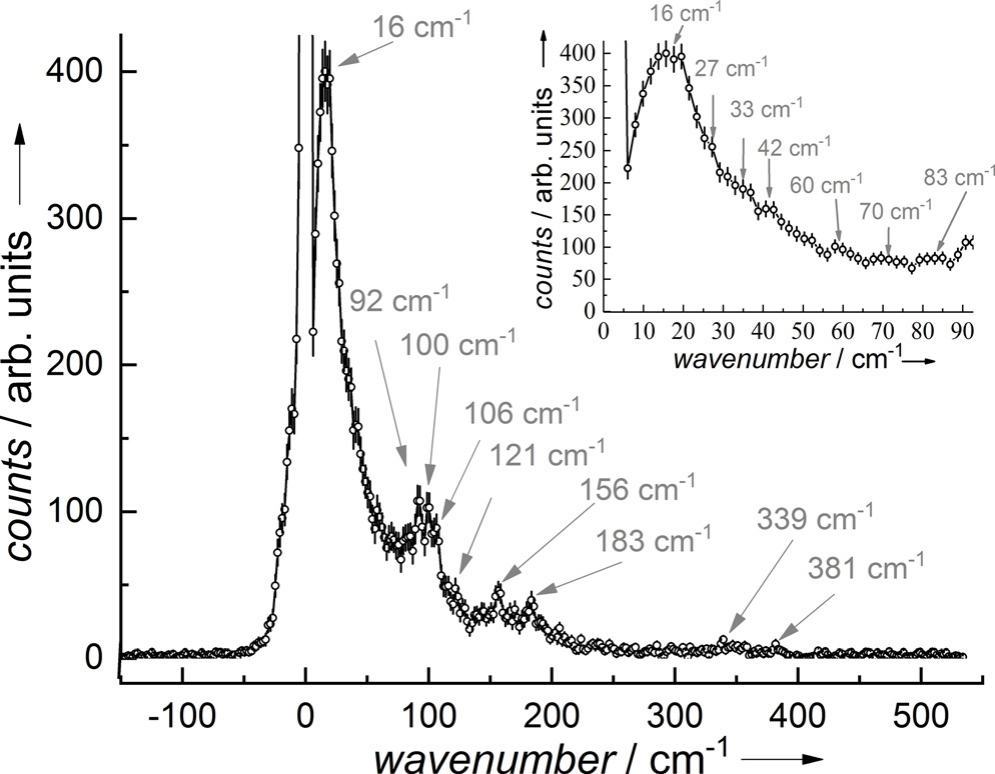
^161^Dy NRVS data of **1** recorded at *T=*21(3) K showing a vanishing annihilation part and a pronounced phonon creation part composed of several peaks as indicated with the arrows showing the maxima of the peak positions. The inset shows the low‐frequency bands that can be assigned to intramolecular vibrations, see Figure 3 and the Supporting Information.

Figure 3 shows the experimental pDOS derived from the ^161^Dy NRVS data recorded at *T=*21(3) K shown in Figure 2 (for details in terms of pDOS determination see the Supporting Information). In order to get insights regarding the molecular vibrations of **1**, we performed first preliminary density functional theory (DFT) calculations using Gaussian 16^25^ on the central complex molecule of **1** (B3LYP‐D3/CEP‐31G; see the Supporting Information). After optimization of the molecule's structure (see Table S1 in the Supporting Information), a normal‐mode analysis was carried out and those vibrational modes involving Dy displacements were considered in order to simulate the pDOS^26^ (see Figure 3). The simulated pDOS is in good agreement with the experimentally obtained one, allowing the assignment of the individual bands to vibrational modes (see Figure 3 and Table S2 in the Supporting Information).

**Figure 3:**
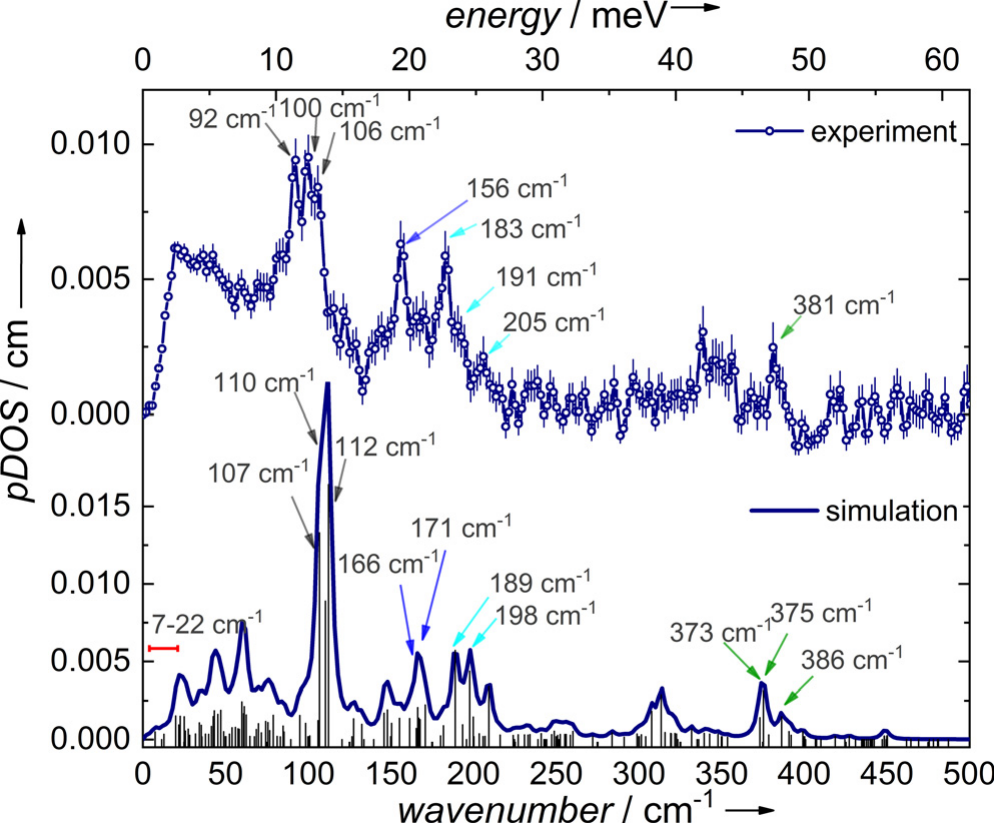
Experimental pDOS of **1** at *T=*21(3) K (top) obtained from the ^161^Dy NRVS data shown in Figure 2 and simulated pDOS using DFT calculations (bottom). The vertical black lines in the simulated data show the energy‐dependent quadratic displacement of the Dy^III^ ion. The differently colored arrows indicate the assignment of modes between experimental and calculated pDOS.

Low‐frequency modes are calculated in the normal‐mode analysis at 7 cm^−1^, 20 cm^−1^, 23 cm^−1^, and 25 cm^−1^, which relate to the experimentally observed low‐frequency peak pattern in the ^161^Dy NRVS data (see inset in Figure 2 and Figure S1). All these low‐frequency modes show a movement of the central Dy‐O_7_ unit, and rotational‐like movement of the Cy_3_PO groups (see for example, Movie 7 in the Supporting Information).

In contrast, the most intense band in the experimental pDOS (100 cm^−1^) relates to three vibrations showing a complete distortion (bending) of the inner coordination shell (Dy‐H_2_O bending). The Dy movement takes place in the equatorial plane of the five coordinated water ligands that move within and perpendicular to this plane.

A different bending‐type of deformation appears for example at 189 cm^−1^ in the simulated pDOS. This mode involves the combined effect of Dy–H_2_O bending (effectively, the five H_2_O molecules move in‐phase along the Dy‐O(‐P) bond) and P‐O‐Dy bending. This results in an effective Dy amplitude along the Dy‐O(‐P) bonds. Further modes with a similar Dy displacement are observable ranging from lower amplitude of Dy‐O(‐P) bending (166 cm^−1^) through additional movement of cyclohexyl rings (171 cm^−1^) to combined water ligand motions in the Dy‐(H_2_O)_5_ plane (198 cm^−1^). The higher energy region (>300 cm^−1^) exhibits stretching‐type modes, involving displacements of the Dy and the five H_2_O molecules in their plane (373 cm^−1^ and 375 cm^−1^) and with additional Dy‐O(P) stretching at 386 cm^−1^.

Considering the approximate *D*_5*h*_ symmetry, most of the Dy displacements occur along the principal symmetry axis and in the equatorial plane. It is noteworthy that the main anisotropy axis of the ground‐state Kramers doublet of **1** is calculated to be almost collinear to this symmetry axis, whereas the one of the first excited state lies in the plane of the five water molecules.^15^ The energy separating the ground‐state and first excited Kramers doublets of **1** was theoretically calculated as 250 cm^−1^, corresponding to magnetic moments of 10 μ_B_ and 1 μ_B_, respectively. The second excited state lies at 276 cm^−1^ with 9 μ_B_.^15^ A comparison of the electronic transition energies with the extracted vibrational modes in this energy region yields vibrations with a negligible amplitude. Thus, we conclude that no relaxation process occurs involving electronic transitions in resonance with molecular vibrations having prominent Dy displacements. However, the detection of such spin‐phonon coupling effects, may be indeed possible when ^161^Dy NRVS is performed in various external magnetic fields in analogy to recently reported field‐dependent Raman and far‐IR spectroscopy studies.^12^, ^13^

Since the magnetic relaxation rate increases with temperature, we wondered whether it is possible to drive vibrational modes into resonance via temperature‐dependent spin‐phonon coupling. Our recent report using ^161^Dy time‐domain synchrotron Mössbauer spectroscopy on compound **1** showed the progressive increase of the relaxation rate especially observable above 55 K, resulting in a faster relaxation at 89(3) K, lying in the range of the Larmor precession time of 1 ns.^27^ Upon increasing temperature, the basic pattern of the pDOS with its three main peak regions is retained (highlighted regions in Figure 4 and Figure S2 for raw data). However, some changes in the position, shape, and the intensity of bands can be observed (see Figure 4). For example, the broadened band at 100 cm^−1^ narrows with increasing temperature accompanied by shifts in the maxima of the peaks. Moreover, the intensity of the band at 156 cm^−1^ almost disappears into the background of the pDOS at both 50 K and 89 K (further details in Figure S3).

**Figure 4:**
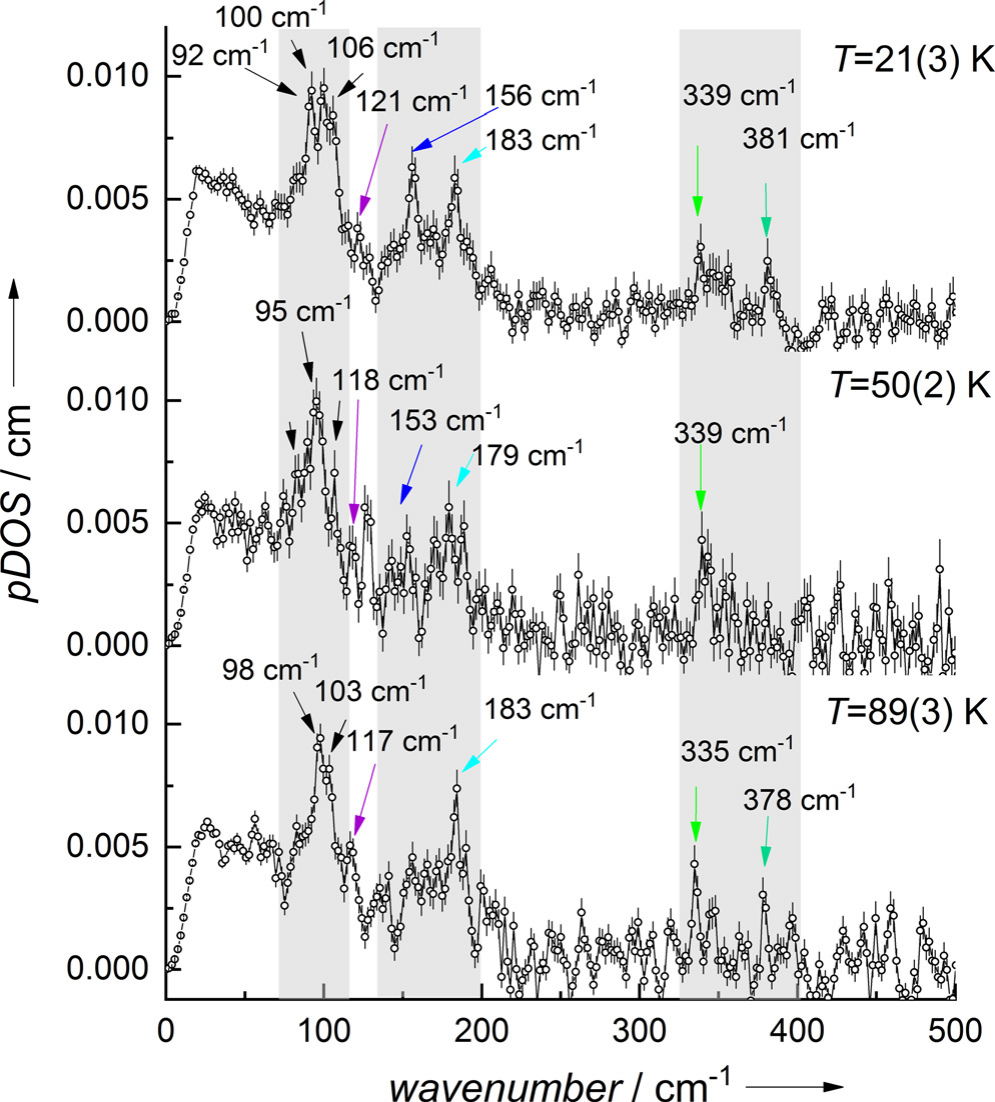
^161^Dy‐pDOS of **1** recorded at indicated temperatures. The regions exhibiting the main vibrational bands are marked in gray. Several maxima are indicated with arrows in order to show the influence of the temperature change (see Figure S3 for enlarged view).

Whereas the ^161^Dy NRVS data are expected to be temperature‐dependent due to multiphonon contributions, the pDOS in the harmonic approximation is assumed to be not affected by temperature. It solely presents a property of the molecular bonds and crystalline lattice.^28^ However, the existence of phase transitions or deviations from the harmonic model are known to influence the pDOS, reflected by modifications of energetic positions or line shapes.^28^, ^29^

A way to account for anharmonic effects in molecular vibrations is to add correction terms to the harmonic frequency of a given mode to account for the coupling of the given mode to the bath of all other modes (see the Supporting Information).^30^ The gradual thermal population probability of low‐energy modes below that of the considered one usually results in band shifts to lower frequencies^31^ with increasing temperature, though reverse shifts are also possible.^32^ Thus, the observed irregular (positive or negative) frequency shifts of specific modes, shown in Figure 4, may be due to the onset of anharmonicity with increasing temperature.

The mean force constant *D*, which can be related to the stiffness of the Dy‐ligand bonds,^17^, ^33^ is one parameter that can be gathered from the analysis of the pDOS (see Table S3 in the Supporting Information).^18^, ^34^ Its magnitude is with *D*=212(25) N m^−1^ interestingly comparable to that reported for intermetallic DyFe_3_, where it describes the hardening of the lattice.^35^ This parameter is temperature independent within experimental error, as expected for the assumed harmonic approximation.

In summary, ^161^Dy NRVS in combination with theoretical DFT simulations gives unique access to optical phonons in SMMs. Considering the unique sensitivity of NRVS to those modes with Dy‐ligand displacement^17^ and the metal center as the decisive spin‐carrier, this technique is perfectly suited to identify those modes that might, via spin‐phonon/vibration coupling, be crucial for spin relaxation. Using this technique in combination with recently developed theoretical ab initio approaches^8^ could lead to important insights regarding spin‐phonon coupling and spin dynamics in SMMs and their relation to inter‐ and intramolecular vibrations.^36^

## Conflict of interest

The authors declare no conflict of interest.

## Supporting information

As a service to our authors and readers, this journal provides supporting information supplied by the authors. Such materials are peer reviewed and may be re‐organized for online delivery, but are not copy‐edited or typeset. Technical support issues arising from supporting information (other than missing files) should be addressed to the authors.

Supplementary

Supplementary
