# Supplementary material for: Exploring the Vibrational Side of Spin‐Phonon Coupling in Single‐Molecule Magnets via 161Dy Nuclear Resonance Vibrational Spectroscopy
Source: Angew Chem Int Ed Engl. 2020 Apr 24;59(23):8818–22. doi: 10.1002/anie.201914728 (PMC7317570; doi:10.1002/anie.201914728)
Supplement: Supplementary file 1 — Supplementary [file ANIE-59-8818-s001.pdf]

Angewandte Chemie
Eine Zeitschrift der Gesellschaft Deutscher Chemiker

Supporting Information

Exploring the Vibrational Side of Spin-Phonon Coupling in Single-Molecule Magnets via ^{161}Dy Nuclear Resonance Vibrational Spectroscopy

Lena Scherthan, Rouven F. Pfleger, Hendrik Auerbach, Tim Hochdörffer, Juliusz A. Wolny, Wenli Bi, Jiyong Zhao, Michael Y. Hu, E. Ercan Alp, Christopher E. Anson, Rolf Diller, Annie K. Powell,* and Volker Schünemann**

anie_201914728_sm_miscellaneous_information.pdf

Table of Contents

Experimental Procedures 3
Experimental Section 3
Synthesis of compound 1 3
Data analysis 3
Results and Discussion 4
Results of density functional theory calculation 4
Low-frequency vibrational modes 5
Temperature-dependent NRVS data 5
Temperature-dependence of thermodynamic parameters and the Lamb-Mößbauer-factor 7
Author Contributions 7

Experimental Procedures

Experimental Section

The Advanced Photon Source at Argonne National Laboratory was run in the standard operating mode (top-up) with a bunch separation of 153 ns. The experiments were carried out at the undulator beamline 3ID by use of the monochromator setup for the ^{161}Dy nuclear resonance (25.651 keV)[1,2]. In order to achieve maximum NRVS count rate at lowest possible sample temperature, the experiments were carried out by utilization of a compact liquid helium flow cryostat, originally designed for high-pressure experiments allowing a minimum base temperature of 9 K. It turned out that this corresponds to a sample temperature of about 20 K (see data analysis), a lower temperature could not be reached with the current available experimental setups at APS. The sample, pressed into a small-sized cylindrical pellet (diameter 600 µm, length 1 mm), was fixed with Apiezon N Cryogenic grease between the diamond culets in the miniature panoramic diamond anvil cell (mini-pDAC).[3] It has to be emphasized that no pressure was applied and that the setup was only chosen to allow adjustment of low sample's temperatures, done by variation of He flow and use of two heaters. The NRVS scans were recorded with two Avalanche Photo Diodes placed outside the cryostat and performing at ambient temperature. The energy of the incoming synchrotron beam, focused to 20 µm, was varied by -30 meV to 70 meV around the nuclear resonance transition energy. The herein presented NRVS spectra result from the sum of 13-18 scans per temperature, with each scan being recorded with a step size of 0.25 meV and a data acquisition time of 3 sec/point. With consideration of the energy resolution of 0.6 meV (≈5 cm^{-1}) and owing to statistical reasons, an uncertainty of 10 % for the energy position is assumed.[1]

Synthesis of compound 1

General procedure: The $^{161}Dy_2O_3$ starting material was obtained from Cyclotron Instrument, Mainz, Germany. The reagents used were commercially available and used without further purification. The powder XRD pattern was recorded on a Stoe STADI-P, using Cu-Kα radiation. For the simulation of the powder pattern the software Diamond 4.4.1 was used[4].

Synthesis: **[$^{161}Dy(Cy_3PO)_2(H_2O)_5$] Br_3·2Cy_3PO·2H_2O·2EtOH** was synthesised by a modification of the literature procedure[5,6], to avoid having first to convert the $^{161}Dy_2O_3$ starting material into $^{161}DyBr_3$. 30 mg (0.811 mmol) of $^{161}Dy_2O_3$ was stirred with 4 ml of deionised water. Hydrochloric acid (10% aq) was added dropwise until the solid was completely dissolved. A saturated aqueous solution of NaOH was added until no more white solid was precipitated. The cloudy solution was centrifuged and the liquid was decanted off. The white solid was washed three times with deionised water, and was then resuspended in 1 ml deionised water. After addition of 58.5 µl (87.2 mg, 0.517 mmol) of a 48% aqueous solution of HBr followed by stirring for 1h, a clear pale orange solution was obtained. To this 4 ml EtOH and 97 mg (0.324 mmol) tricyclohexylphosphine oxide were added while stirring. After filtration, slow evaporation of the solution yielded clear block shaped crystals after one day. The solution was decanted and the crystals were washed with ethanol. Powder XRD confirmed the phase purity of the product by comparison with the calculated powder pattern.

Data analysis

The herein presented pDOS are calculated using the free available software PHOENIX[7], taking the resonance energy of 25.651 keV and recoil energy of 2.195 meV into account.[8] The data evaluation procedure determines and removes the elastic contribution from the NRVS data, separates the single-phonon absorption from multiphonon contributions based on a harmonic lattice model.[7]

The NRVS data are composed of a vanishing negative energy (phonon annihilation, anti-Stokes) part and a positive energy (phonon creation, Stokes) region showing several frequency bands. This overall asymmetric structure is related to the law of detailed balance, since the phonon annihilation depends on the phonon occupation number, being negligible at low temperatures.[9] The elastic resonant absorption without annihilation or creation of phonons leads to the resonant peak at zero energy (elastic peak).[10] Consequently, based on the detailed balance principle, the sample's temperature is accessible from the NRVS data (see Table S3). However, it has to be considered that this procedure is not highly accurate in this low-temperature region owing to the vanishing annihilation part.[11] Owing to the experimental setup using a compact liquid helium flow cryostat, the sample temperature is generally higher than the three selected preset temperature values of T_{set}=10 K, T_{set}=40 K, T_{set}=80 K (compare to Table 1).

Next to the pDOS calculation, the analysis with PHOENIX includes the calculation of the thermodynamic parameters and the Lamb-Mössbauer factor[7], as described in detail in [11,12]. Following the notation given in [11], the extraction of the parameters is based on the *i*th moment M_i^g of the pDOS $g(E)$ or on the thermalized moment T_i^g

$$M_i^g = \int_0^\infty E^i \, g(E) dE$$

$$T_i^g = \int_0^\infty \frac{1}{2} \coth(\frac{E}{2k_B T}) E^i \, g(E) dE$$

Using these relations, information about the Lamb-Mössbauer factor can be gathered, considering the following relation to the mean square displacement $\langle x^2 \rangle$

$$f_{LM} = \exp(-k^2 \langle x^2 \rangle)$$

$$\langle x^2 \rangle = \frac{\hbar^2}{M} T_{-1}^g$$

where k denotes the wavenumber related to the nuclear resonance transition and M is the mass of ^{161}Dy. Assuming a harmonic lattice potential, the mean force constant is related to the second moment of the pDOS via

$$D = \frac{M}{\hbar^2} M_2^g$$

Results and Discussion

Results of density functional theory calculation

DFT calculations were performed with the Gaussian 16[13] programme using the B3LYP[14] functional and CEP-31G basis set[15]. Grimme D3 dispersion corrections were applied [16]. The complex molecule of $[Dy(Cy_3PO)_2(H_2O)_5]Br_3$ was optimized, starting from the X-ray structure[5] . The subsequent calculation of normal modes revealed no imaginary frequencies.

Table S1: Experimental and calculated principal bonds with interatomic distances (in Å) and bond angles (in degree) for the title molecule of compound **1**, showing that the optimized structure is in reasonable agreement with the X-ray structure. [5]

	**bond length Å**		**bond length Å**					**distance Å**			**angle (°)**	**angle (°)**				
	**Dy-O(P)**		**Dy-O(H_2O)**					**Dy-Br**			**O(P)-Dy-O(P)**	**H_2O-Dy-OH_2**				
**DFT**	2.189	2.194	2.409	2.375	2.392	2.398	2.358	4.817	4.835	4.809	179	73	72.5	69	72	74
**X-ray**	2.210	2.190	2.344	2.355	2.362	2.336	2.365	4.833	4.814	4.765	179	70.7	71.4	72.0	72.8	73.3

Table S2: Vibrational modes including Dy displacement as obtained by normal mode analysis for optimized structure of **1**. Movies for all the modes are additionally given as ESI. The names of the individual movie files correspond to the frequencies of the modes, as listed here.The symmetry (symm.) of the vibrations was obtained by comparison of the discussed modes with those of IF_7 representing the D_{5h} symmetry, assuming the same ideal symmetry for the idealised coordination core of the complex under study.

Mode (cm^{-1})	Symm.	Exp. (cm^{-1})	Classification of vibrational mode including Dy displacement
7<br>20<br>22<br>23<br>25	--	16	movement of the central Dy-O_7 unit, and rotational-like movement of the Cy_3PO groups and a strong bromine amplitude (that at 7 cm^{-1}), wing-like movement of the cyclohexyl ring (these at 7, 20 and 22 cm^{-1}) and rotation of the cyclohexyl ring around the P-C-ring (modes at 23 and 25 cm^{-1}).
43<br>45<br>47	--	43	Minor amplitude of Dy movement, but pronounced rotation of the cyclohexyl ring around the P-C-ring and the P-O-Dy bending
60<br>61<br>61<br>62	--	60	Minor amplitude of Dy movement, pronounced rotation of the cyclohexyl ring around the P-C-ring and the P-O-Dy bending
107<br>110<br>112	E_1'	92<br>100<br>106	H_2O-Dy-OH_2 bending and Dy movement mainly in the Dy(H_2O)$_5$ plane. Particularly strong amplitude of Dy movement in this plane for the mode at 112 cm^{-1}.
127		121	Similar to those predicted at 107-112 cm^{-1}.
146<br>148	--	144	Similar to those predicted at 107-112 cm^{-1}. Water ligands movement occurs mainly perpendicular to the equatorial plane (flipping).
166<br>167<br>168<br>171	A_2''<br>--<br><br>A_2''	156	Similar to those predicted at 107-112 cm^{-1}. With a significant amplitude of the Dy displacement perpendicularly to the equatorial plane and some P-O-Dy bending. Mode at 171 cm^{-1} includes movement of cyclohexyl rings
189<br>198	A_2''<br>A_2''	183	Analogous to mode at 166 cm^{-1}, with higher amplitude of the in-equatorial plane movement of Dy.<br>Analogous to 189 cm^{-1} with water ligands moving in Dy-water plane
200	--	191	Similar to those predicted at 189 and 198 cm^{-1}
209	--	205	Similar to those predicted at 189 and 198 cm^{-1}
308<br>313	--	339-356	Stretching modes with displacement of Dy. For the mode at 308 cm^{-1} the O(P) stretching dominates, while for the that 313 cm^{-1} the Dy-O(water) stretching is pronounced.
373<br>375<br>386	E_1'<br>E_2'<br>--	381	Stretching type modes with displacement of Dy and five water molecules in the Dy-(H_2O)$_5$-plane (at 373 and 375 cm^{-1}) with additional Dy-O(P) stretching (at 386 cm^{-1})

Low-frequency vibrational modes

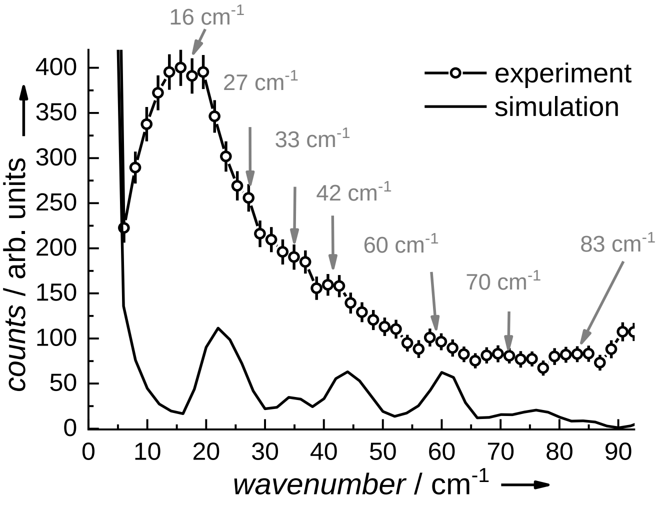

Figure S1: Low-frequency part of the NRVS spectra recorded at a sample temperature of 21(3) K. The comparison with the simulated NRVS spectrum based on the normal mode analysis shows the existence of intramolecular vibrational modes that can be assigned to the experimentally observed peak structure.

It should be noted that the low-energy part in the experimental data, that can be slightly affected by the subtraction of the elastic peak during data reduction procedure[10], can be mostly assigned to acoustic modes, but is occasionally also populated by optical modes.[17] The estimation of the contribution of the acoustic modes is based on the fraction of the kinetic energy associated with the motion of the Mössbauer atom in each respective mode, the so-called mode composition factors[18]. This formalism represents the basis for the calculation of the pDOS from the normal modes[18,19]. The calculation is further based on the numerical integration of the experimental data and assumes that the inter- and intramolecular modes are independent of each other[17,18]. Taking the relative mass of Dy compared to the total molecular mass of **1** into account, this energetic limit of the region of acoustic modes is estimated as 30 cm^{-1}.

Temperature-dependent NRVS data

As a second step, we have investigated the influence of the temperature on the vibrational properties of the SMM **1**. With increasing temperature, the NRVS data is dominantly changed near the resonant peak, showing a rise in the negative wing of the spectrum with a distinct phonon peak (see Fig. S2, marked with arrows). As mentioned before, this increase in intensity can be attributed to the thermal occupation of low-lying phonon modes.[9,20] A further temperature-dependent effect influencing the NRVS spectra results from the increasing probability of multi-phonon contributions. However, these terms are eliminated by calculation of the pDOS (see Data Analysis section).

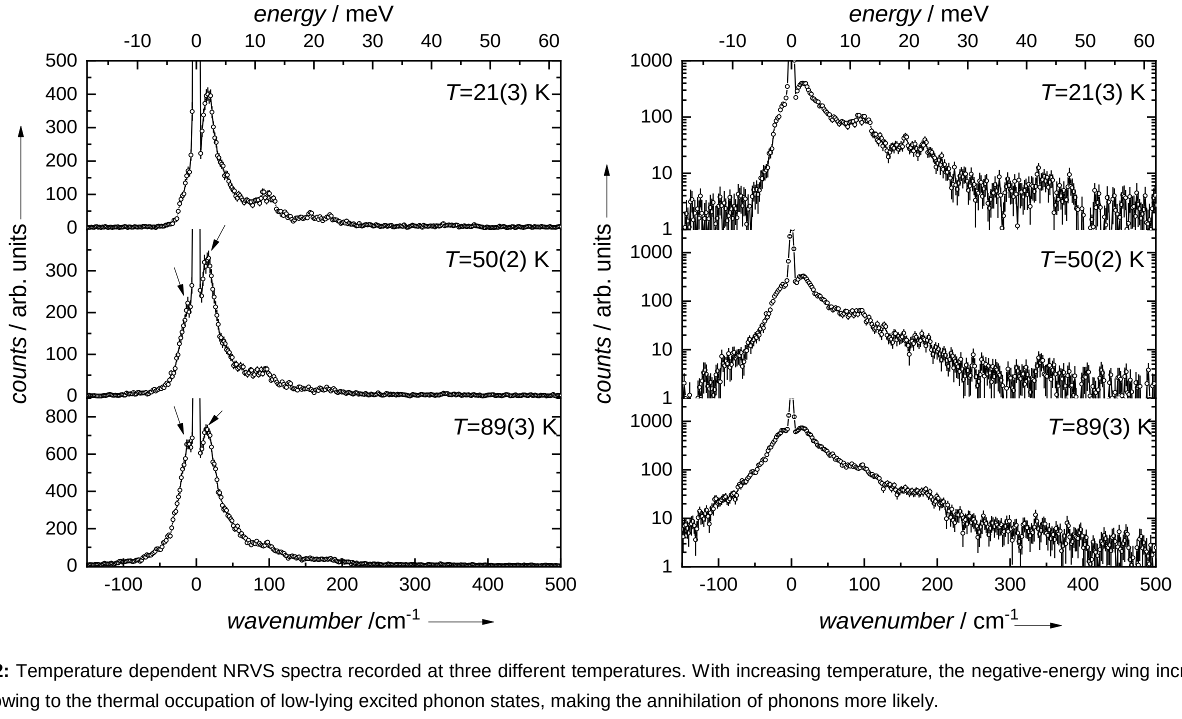

Figure S2: Temperature dependent NRVS spectra recorded at three different temperatures. With increasing temperature, the negative-energy wing increases in intensity owing to the thermal occupation of low-lying excited phonon states, making the annihilation of phonons more likely.

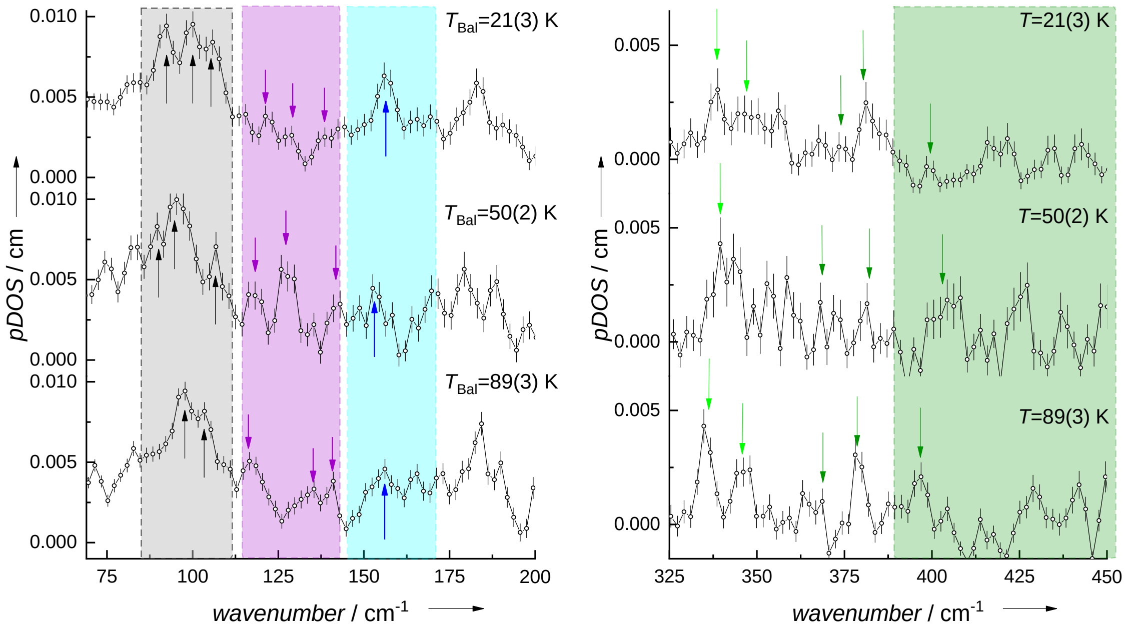

Figure S3: Enlarged view of selected energetic parts of the experimental pDOS of **1**. The colored parts are those showing the most pronounced modifications upon the temperature change (compare Figure S4, also mentioned in the description in the main text).

Figure S3 highlights further spectral changes, which occur, for example, also in the range 115-145 cm^{-1} (see Figure S3, gray and purple background pattern). From the simulated pDOS, most of these observed frequency bands could be assigned to several intramolecular vibrations that are separated by only 2-3 cm^{-1}. Therefore, based on the current data we consider it as not reliable to allocate the experimentally observed changes to certain distinct calculated vibrational modes.

Discussion of spin-phonon coupling and anharmonic effects

In general, anharmonicity describes the existence of non-quadratic terms in the inter-atomic potentials, as e. g. by non-symmetric potentials[21], or via spin-phonon coupling.[22] As outlined before, spin-phonon coupling is indeed assumed to be one key mechanism related to spin-dynamics in SMMs.[23,24] The frequently employed Debye-approach as a spin-phonon model for molecular compounds has however nowadays become obsolete.[23,24] Firstly, the restriction on acoustic phonons and secondly the assumption of a uniform spin-phonon coupling intensity is not appropriate for SMMs.[25,26] This has been shown just recently by independently reported ab initio spin dynamic approaches emphasizing the importance of the coupling strength of acoustic and especially optical intramolecular vibrational modes including the distortion of the coordination environment of the central metal center.[24,25,27] The therein presented models include anharmonic force constants, revealing a temperature dependency of anharmonic effects and the responsibility for under-barrier relaxation at high temperatures.[26,27]

One way to account for anharmonic effects in molecular vibrations is to add correction terms to the harmonic frequency of a given mode. The correction terms are determined by (diagonal and off-diagonal) anharmonic coefficients that represent the coupling of the given mode to the bath of all other modes. The anharmonic coefficients can be derived from the cubic and quartic force constants in a normal coordinate basis and are negative in most cases, leading to a decrease of the given (harmonic) frequency. The band shift with increasing temperature is then determined by the anharmonic coefficients, the (thermal) population probability of the modes with energies below that of the considered one, and the method-specific line shape function.[28]
At higher temperature (e.g. $T \gtrsim 300$ K) and higher frequencies (e.g. $\gtrsim 800$ cm^{-1}) for a molecule of this size this usually results (via "averaging" over a larger number of lower-energy populated modes with mostly negative anharmonic coefficients) in a systematic red-shift of the observed vibrational frequency with increasing temperature, together with asymmetric line broadening.[29] A recent computational study of a transition metal complex has also proven the occurrence of negative frequency shifts for specific bands.[30] As shown in Fig. 4 (spectral regions marked in grey), the changes observed for the pDOS of **1** are not systematic, neither regarding band position nor band shape. However, this finding is still conceivable within the framework described above. Here, at low temperature and small frequencies, for a given mode the number of effectively coupled lower-energy bath modes is strongly reduced. Consequently, the gradual population of the low-energy bath modes, and thus the switching-on of the respective anharmonicity with increasing temperature, might favor the relative impact of specific modes with positive (or, in other cases or at another temperature negative) anharmonic coefficients. This, in turn, could lead to the observed irregular (positive or negative) frequency shifts.

In the context of anharmonic effects, there is one further effect that should be mentioned. In the case of the Fe-based SMM $[(tpa^{PH})Fe]^{-}$, the presence of a lowest phonon mode close to twice the energy of the effective energy barrier was reported by inclusion of anharmonicity.[26] However, such a relation between the vibrational energies and the high effective energy barrier of **1** (543(2) K; 377 cm^{-1} [5]) is not observable within this study since the vibrational modes including ^{161}Dy displacements are located in a lower energy region (<400 cm^{-1}).

Temperature-dependence of thermodynamic parameters and the Lamb-Mößbauer-factor

Table S3. Lamb-Mößbauer factor and thermodynamic parameters as deduced from the ^{161}Dy-NRVS data using PHOENIX[7,31]. The parameters are additionally calculated from the simulated pDOS[10,11,32]. The values are obtained using the formalism introduced in the section ‚Data analysis', described on p. 3.

	Experimental pDOS			Simulation
T (K)	21(3)	50(2)	89(3)	21[a]
f_{LM}	0.64(2)	0.47(2)	0.30(2)	0.67
$f_{LM}(T\text{=0 K})$	0.76(1)	0.77(1)	0.77(1)	0.8
$\langle x^2 \rangle$ (Å)	0.0027(3)	0.0045(4)	0.0072(4)	0.0024
E_{Kin} (meV/atom)	3.8(2)	4.3(2)	5.6(1)	4.6
Cv (k_B/atom)	0.60(9)	1.46(5)	2.10(5)	0.40
S_{vib} (k_B/atom)	0.38(9)	1.23(9)	2.12(5)	0.23
D (Nm^{-1})	212(25)	266(40)	263(25)	305

[a] Parameters deduced from theoretically calculated pDOS are obtained by assumption of a temperature-independent pDOS and calculation of parameters with assumption of a temperature of 21 K.

It is worth noting that the Lamb-Mößbauer-factor f_{LM} shows a pronounced decay upon temperature increase (see Figure S4), directly related to the increase of the mean-square displacement $\langle x^2 \rangle$ of the Dy(III) ions (see Table S3). Such a temperature dependence of f_{LM} can be attributed to the increasing thermal population of vibrational states.[33] Furthermore, the kinetic energy E_{kin}, the specific heat capacity C_v and the vibrational entropy E_{vib} show a pronounced temperature dependency.

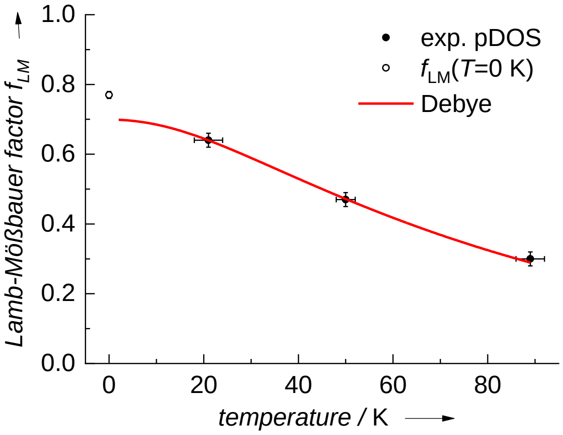

Figure S4: Temperature dependent Lamb-Mößbauer factor as obtained by NRVS data reduction and calculation of pDOS. With increasing temperature, the Lamb-Mössbauer factor decreases. A theoretical analysis of this behavior is often based on the Debye-model[34], which is however grossly inadequate for molecular compounds[34]. An approximated Debye-temperature can be gained by neglecting the zero-energy value calculated out of the thermalized moment of the spectrum[11,32], resulting in a Debye-temperature of about 107 K.

Author Contributions

Conceptualization of the ^{161}Dy-NRVS experiments was carried out by V. S. and L. S.; The SMM was proposed by A. K. P.; W. B., J. Z., M. Y. H. and E. E. A. provided the experimental-setup and supported the experiments, that were performed by L. S., H. A., T. H., W. B. and V. S.; R. F. P, C. E. A. and A. K. P. were responsible for the sample preparation; J. A. W. carried out the DFT calculations; L. S. carried out the data analysis. R. D. contributed to the discussion of anharmonic effects. L. S. and V. S. wrote the paper draft, all authors added their respective contributions, reviewed and edited the paper.

References

[1] D. E. Brown, T. S. Toellner, W. Sturhahn, E. E. Alp, M. Hu, R. Kruk, K. Rogacki, P. C. Canfield, *Hyperfine Interact.* **2004**, *153*, 17.
[2] T. S. Toellner, M. Y. Hu, G. Bortel, W. Sturhahn, D. Shu, *Nucl. Instr. Meth. Phys. Res. A* **2006**, *557*, 670.
[3] J. Y. Zhao, W. Bi, S. Sinogeikin, M. Y. Hu, E. E. Alp, X. C. Wang, C. Q. Jin, J. F. Lin, *Rev. Sci. Instrum.* **2017**, *88*, 125109.
[4] Diamond 4.4.1, Crystal Impact GbR, Bonn, Germany, 2017.
[5] Y.-C. Chen, J.-L. Liu, L. Ungur, J. Liu, Q.-W. Li, L.-F. Wang, Z.-P. Ni, L. F. Chibotaru, X.-M. Chen, M.-L. Tong, *J. Am. Chem. Soc.* **2016**, *138*, 2829.
[6] A. Bowden, A. M.J. Lees, A. W.G. Platt, *Polyhedron* **2015**, *91*, 110.
[7] W. Sturhahn, *Hyperfine Interact.* **2000**, *125*, 149.
[8] Y. V. Shvyd'ko, M. Lucht, E. Gerdau, M. Lerche, E. E. Alp, W. Sturhahn, J. Sutter, T. S. Toellner, *J. Synchrotron Rad.* **2002**, *9*, 17.
[9] A. I. Chumakov, R. Rüffer, O. Leupold, A. Barla, H. Thiess, J. M. Gil, H. V. Alberto, R. C. Vilão, N. Ayres de Campos, V. G. Kohn et al., *Phys. Rev. B* **2001**, *63*, 3828.
[10] V. G. Kohn, A. I. Chumakov, *Hyperfine Interact.* **2000**, *125*, 205.
[11] N. Dauphas, M. Y. Hu, E. M. Baker, J. Hu, F. L. H. Tissot, E. E. Alp, M. Roskosz, J. Zhao, W. Bi, J. Liu et al., *J. Synchrotron Rad.* **2018**, *25*, 1581.
[12] a) M. Y. Hu, W. Sturhahn, T. S. Toellner, P. M. Hession, J. P. Sutter, E. E. Alp, *Nucl. Instrum. Methods Phys. Res. A* **1999**, *428*, 551; b) A. I. Chumakov, W. Sturhahn, *Hyperfine Interact.* **1999**, *123/124*, 781.
[13] Gaussian 16, Revision A.03, M. J. Frisch, G. W. Trucks, H. B. Schlegel, G. E. Scuseria, M. A. Robb, J. R. Cheeseman, G. Scalmani, V. Barone, G. A. Petersson, H. Nakatsuji, X. Li, M. Caricato, A. V. Marenich, J. Bloino, B. G. Janesko, R. Gomperts, B. Mennucci, H. P. Hratchian, J. V. Ortiz, A. F. Izmaylov, J. L. Sonnenberg, D. Williams-Young, F. Ding, F. Lipparini, F. Egidi, J. Goings, B. Peng, A. Petrone, T. Henderson, D. Ranasinghe, V. G. Zakrzewski, J. Gao, N. Rega, G. Zheng, W. Liang, M. Hada, M. Ehara, K. Toyota, R. Fukuda, J. Hasegawa, M. Ishida, T. Nakajima, Y. Honda, O. Kitao, H. Nakai, T. Vreven, K. Throssell, J. A. Montgomery, Jr., J. E. Peralta, F. Ogliaro, M. J. Bearpark, J. J. Heyd, E. N. Brothers, K. N. Kudin,. N. Staroverov, T. A. Keith, R. Kobayashi, J. Normand, K. Raghavachari, A. P. Rendell, J. C. Burant, S. S. Iyengar, Tomasi, M. Cossi, J. M. Millam, M. Klene, C. Adamo, R. Cammi, J. W. Ochterski, R. L. Martin, K. Morokuma, O. Farkas, J. B. Foresman, and D. J. Fox, Gaussian, Inc., Wallingford CT, 2016.
[14] A. D. Becke, *J. Chem. Phys.* **1993**, *98*, 5648.
[15] a) W. J. Stevens, H. Basch, M. Krauss, *J. Chem. Phys.* **1984**, *81*, 6026; b) W. J. Stevens, M. Krauss, H. Basch, P. G. Jasien, *Can. J. Chem.* **1992**, *70*, 612; c) T. R. Cundari, W. J. Stevens, *J. Chem. Phys.* **1993**, *98*, 5555.
[16] S. Grimme, J. Antony, S. Ehrlich, H. Krieg, *J. Chem. Phys.* **2010**, *132*, 154104.
[17] A. I. Chumakov, R. Rüffer, O. Leupold, I. Sergueev, *Struct. Chem.* **2003**, *14*, 109.
[18] J. T. Sage, C. Paxson, G. R. A. Wyllie, W. Sturhahn, S. M. Durbin, P. M. Champion, E. E. Alp, W. R. Scheidt, *J. Phys.: Condens. Matter* **2001**, *13*, 7707.
[19] H. Paulsen, H. Winkler, A. X. Trautwein, H. Grünsteudel, V. Rusanov, H. Toftlund, *Phys. Rev. B* **1999**, *59*, 975.
[20] V. G. Kohn, A. I. Chumakov, R. Rüffer, *Phys. Rev. B* **1998**, *58*, 8437.
[21] A. I. Chumakov, R. Rüffer, *Hyperfine Interact.* **1998**, *113*, 59.
[22] A. Q. R. Baron, *Introduction to High-Resolution Inelastic X-Ray Scattering. revised 2018, http://arxiv.org/abs/1504.01098v5*, **2015**.
[23] J.-L. Liu, Y.-C. Chen, M.-L. Tong, *Chem. Soc. Rev.* **2018**, *47*, 2431.
[24] L. Escalera-Moreno, J. J. Baldoví, A. Gaita-Ariño, E. Coronado, *Chem. Sci.* **2018**, *9*, 3265.
[25] A. Lunghi, F. Totti, S. Sanvito, R. Sessoli, *Chem. Sci.* **2017**, *8*, 6051.
[26] A. Lunghi, F. Totti, R. Sessoli, S. Sanvito, *Nat. Commun.* **2017**, *8*, 14620.
[27] L. Escalera-Moreno, N. Suaud, A. Gaita-Ariño, E. Coronado, *J. Phys. Chem. Lett* **2017**, *8*, 1695.
[28] P. Hamm, S. M. Ohline, W. Zinth, *J. Chem. Phys.* **1997**, *106*, 519.
[29] a) D. Imanbaew, Y. Nosenko, C. Kerner, K. Chevalier, F. Rupp, C. Riehn, W. R. Thiel, R. Diller, *Chem. Phys.* **2014**, *442*, 53; b) M. Zimmer, F. Rupp, P. Singer, F. Walz, F. Breher, W. Klopper, R. Diller, M. Gerhards, *Phys. Chem. Chem. Phys.* **2015**, *17*, 14138.
[30] J. Wu, C. Sousa, C. de Graaf, *Magnetochemistry* **2019**, *5*, 49.
[31] W. Sturhahn, *PHOENIX is free software available via http://www.nrixs.com/conuss.html, Version PHOENIX-2.1.4, July, 07 2019*.
[32] M. Y. Hu, T. S. Toellner, N. Dauphas, E. E. Alp, J. Zhao, *Phys. Rev. B* **2013**, *87*, 64301.
[33] P. Gütlich, H. A. Goodwin, *Spin Crossover in Transition Metal Compounds III*, Springer Berlin Heidelberg, **2004**.
[34] N. N. Greenwood, T. C. Gibb, *Mössbauer Spectroscopy*, Chapman and Hall Ltd., London, **1971**.
