# Supplementary figures and images for: Exploring the Vibrational Side of Spin‐Phonon Coupling in Single‐Molecule Magnets via 161Dy Nuclear Resonance Vibrational Spectroscopy
Source: Angew Chem Int Ed Engl. 2020 Apr 24;59(23):8818–22. doi: 10.1002/anie.201914728 (PMC7317570; doi:10.1002/anie.201914728)

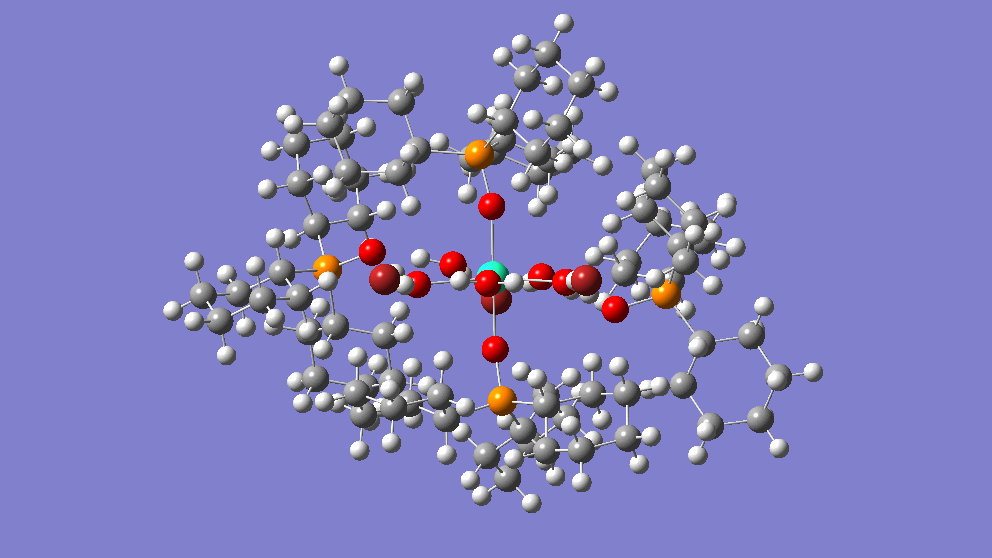

Supplement: Supplementary file 2 — Supplementary [file ANIE-59-8818-s002.zip › 107.gif]

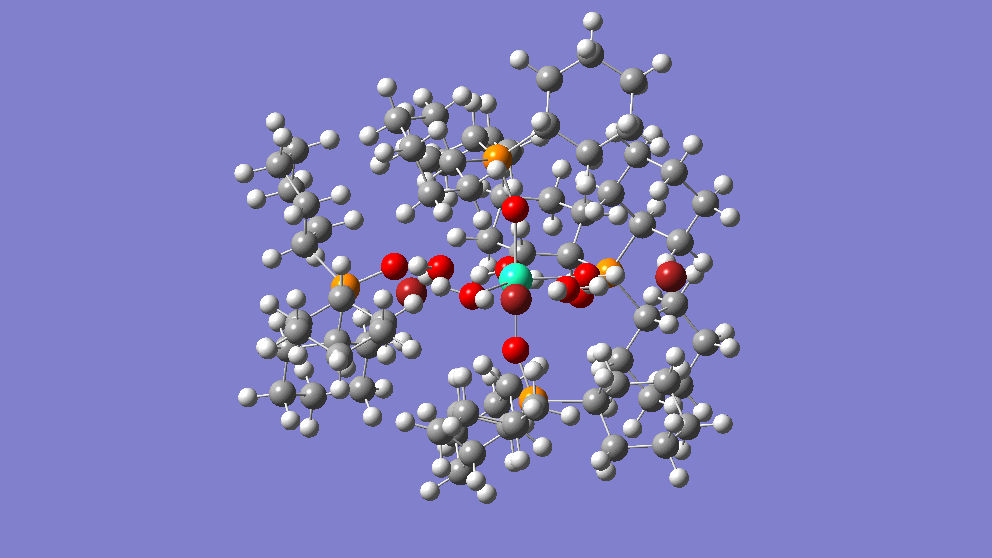

Supplement: Supplementary file 2 — Supplementary [file ANIE-59-8818-s002.zip › 110.gif]

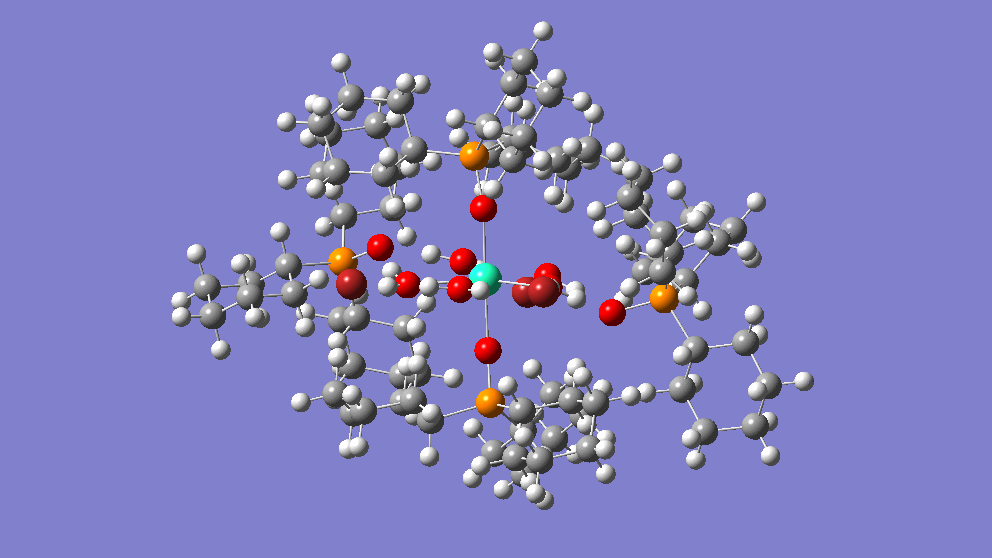

Supplement: Supplementary file 2 — Supplementary [file ANIE-59-8818-s002.zip › 112.gif]

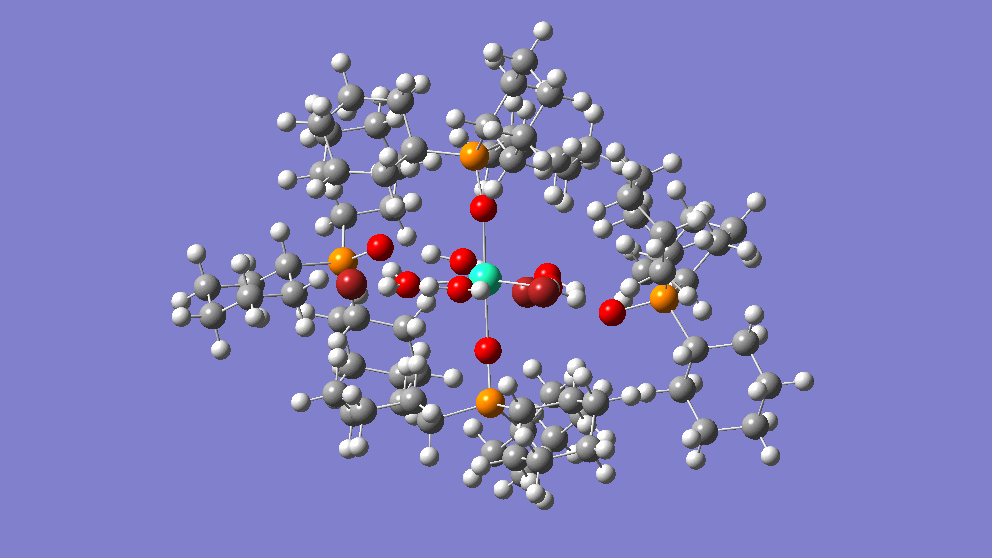

Supplement: Supplementary file 2 — Supplementary [file ANIE-59-8818-s002.zip › 127.gif]

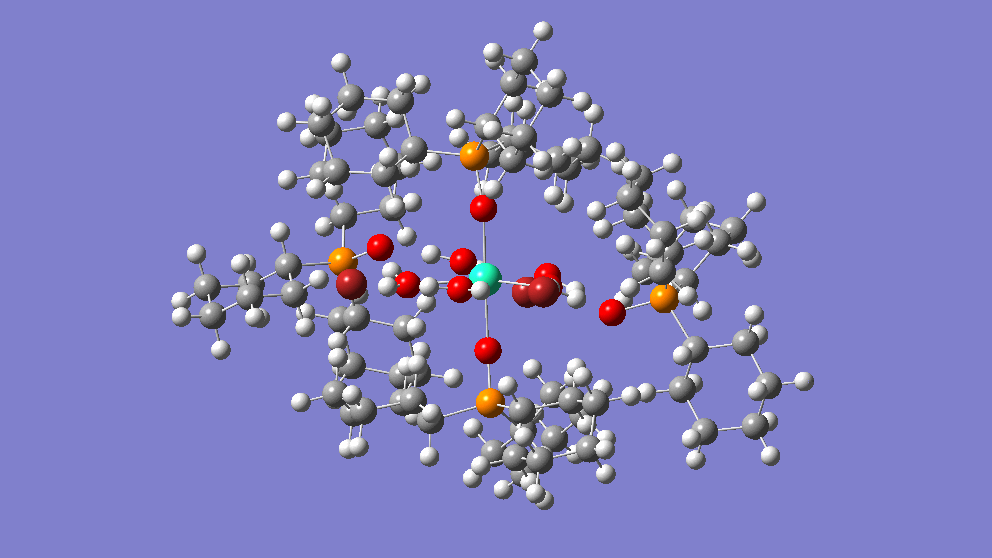

Supplement: Supplementary file 2 — Supplementary [file ANIE-59-8818-s002.zip › 146.gif]

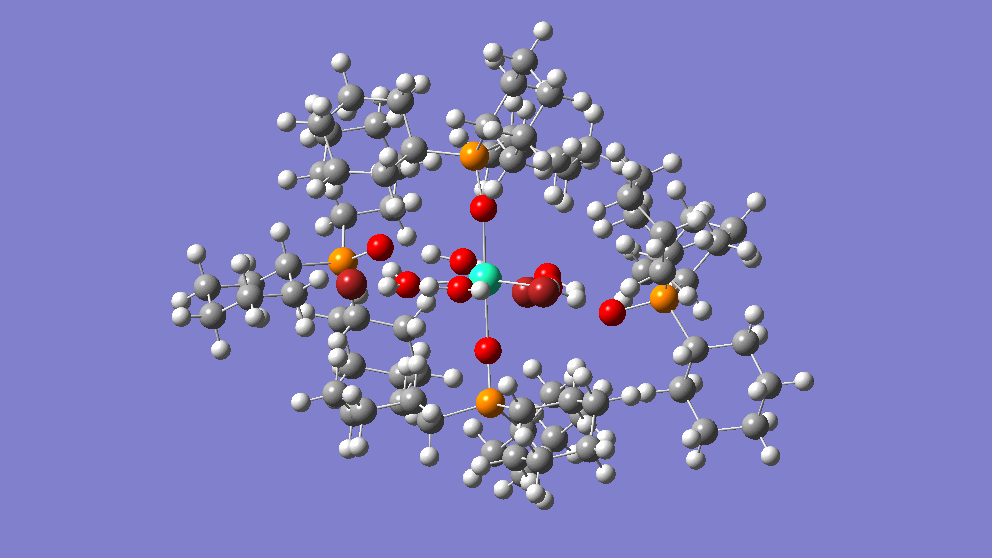

Supplement: Supplementary file 2 — Supplementary [file ANIE-59-8818-s002.zip › 148.gif]

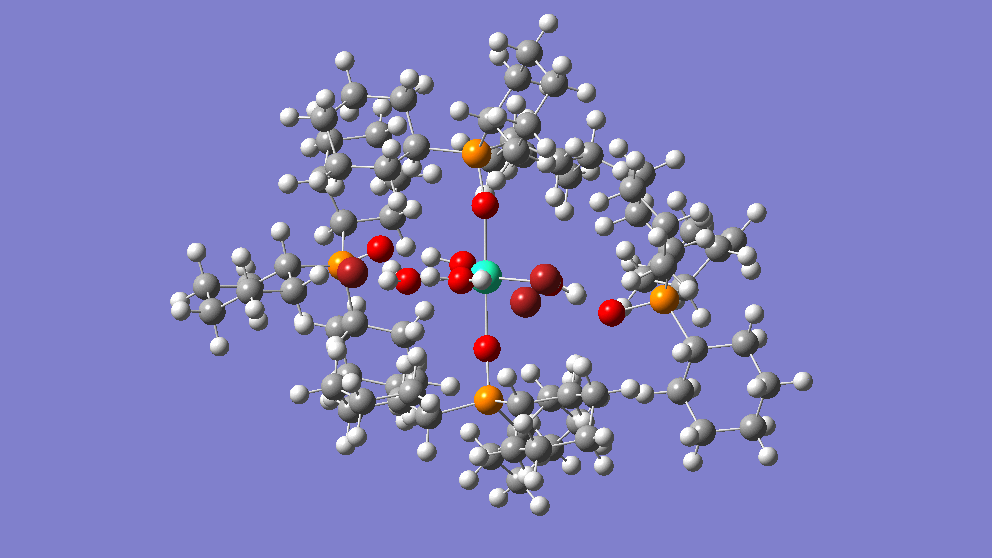

Supplement: Supplementary file 2 — Supplementary [file ANIE-59-8818-s002.zip › 166.gif]

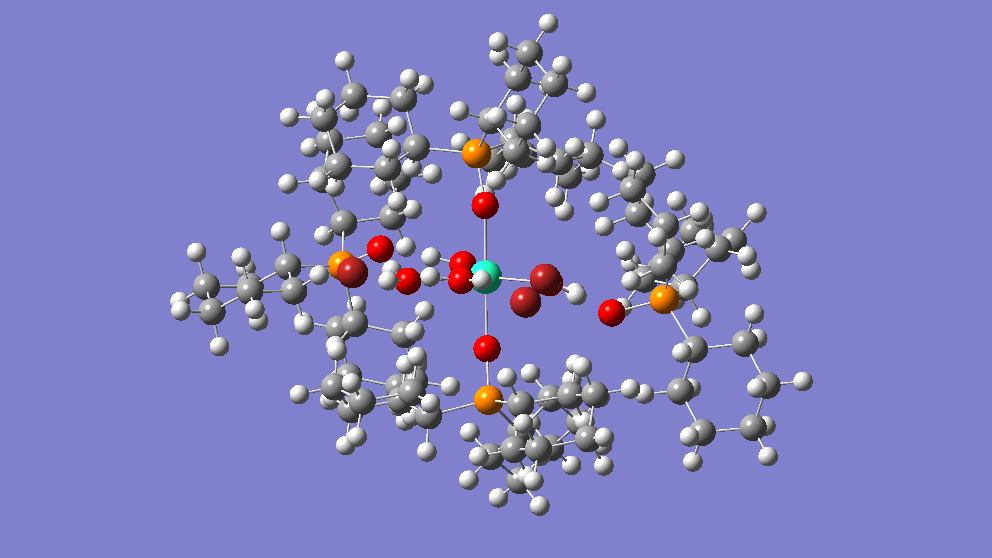

Supplement: Supplementary file 2 — Supplementary [file ANIE-59-8818-s002.zip › 167.gif]

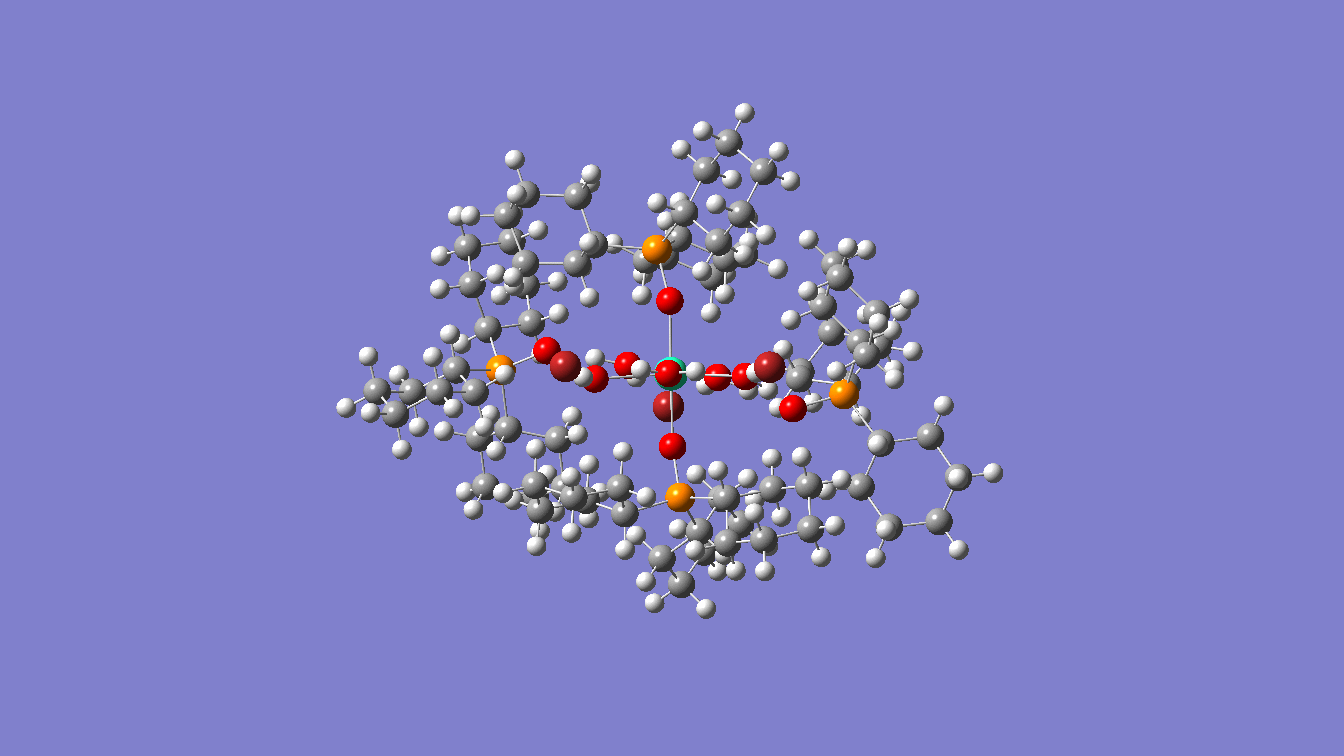

Supplement: Supplementary file 2 — Supplementary [file ANIE-59-8818-s002.zip › 168.gif]

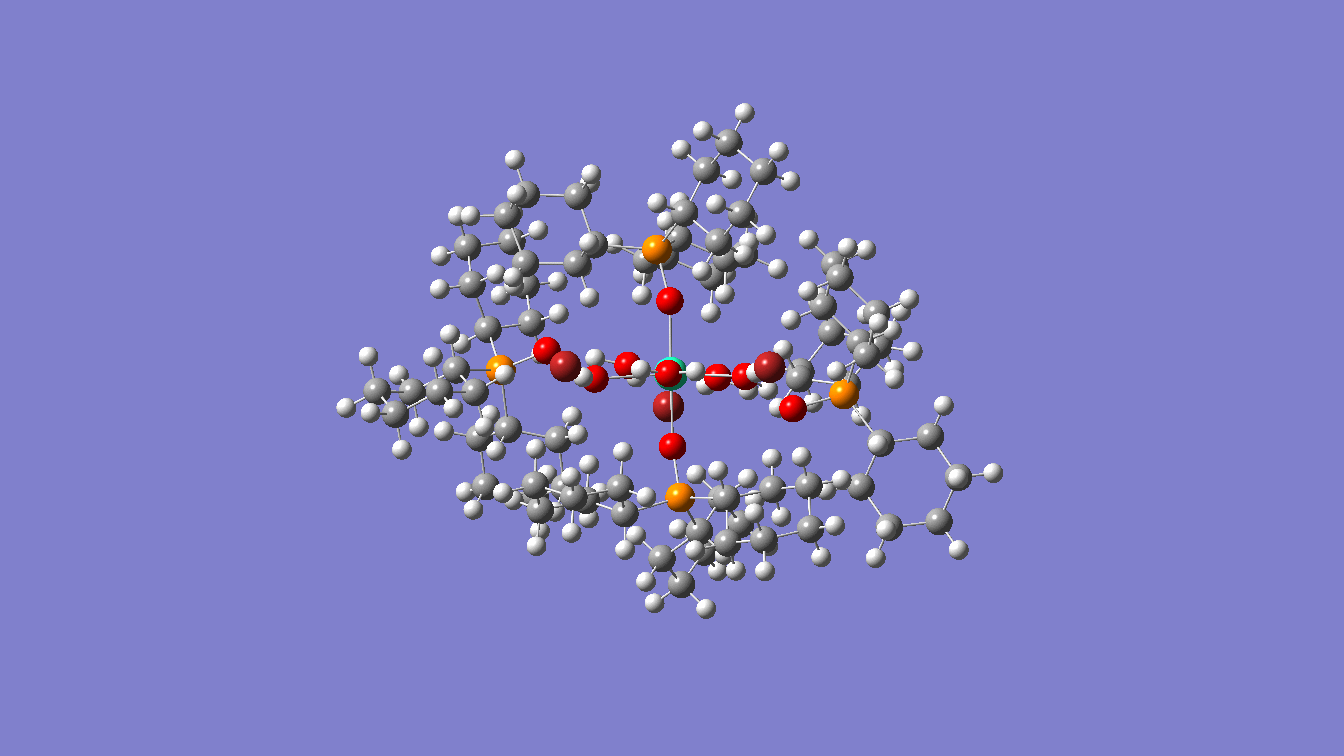

Supplement: Supplementary file 2 — Supplementary [file ANIE-59-8818-s002.zip › 171.gif]

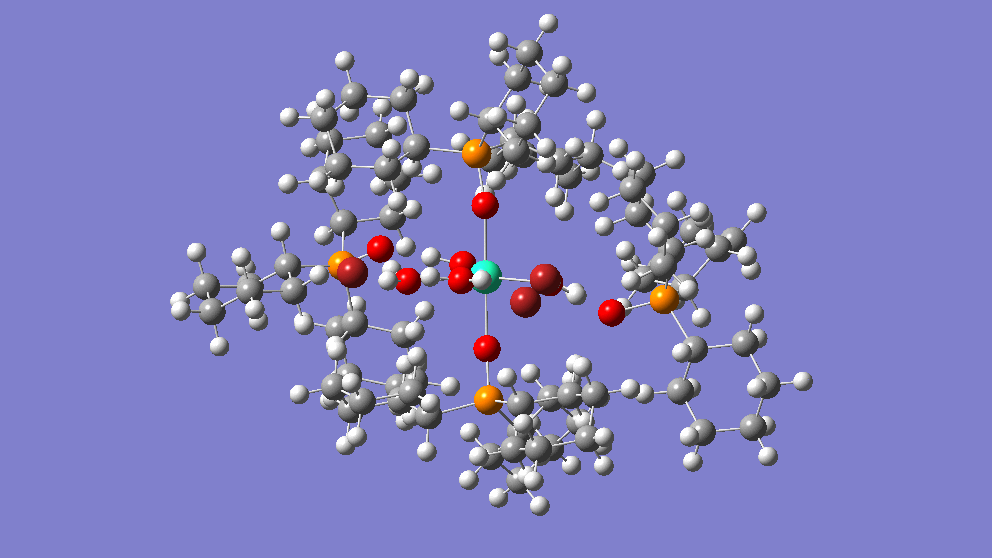

Supplement: Supplementary file 2 — Supplementary [file ANIE-59-8818-s002.zip › 189.gif]

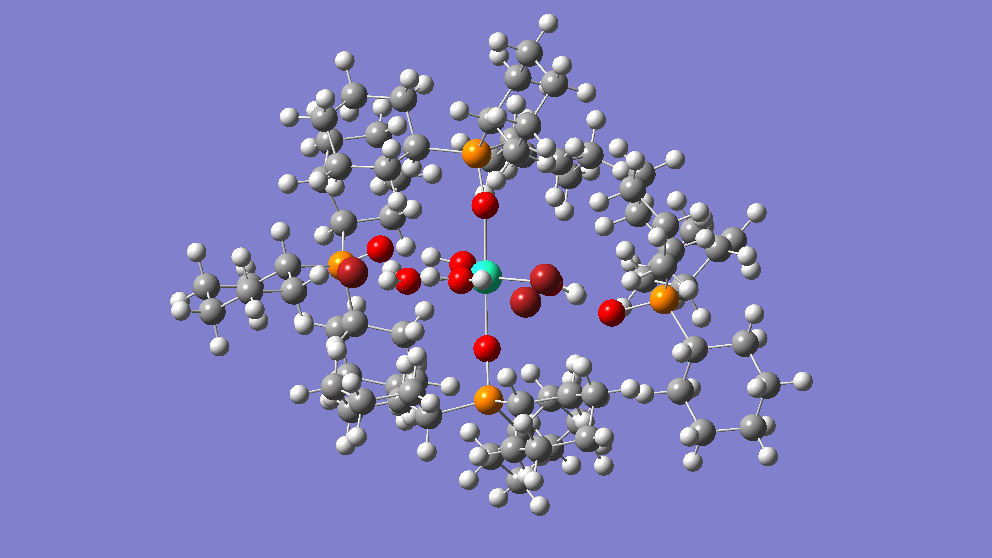

Supplement: Supplementary file 2 — Supplementary [file ANIE-59-8818-s002.zip › 198.gif]

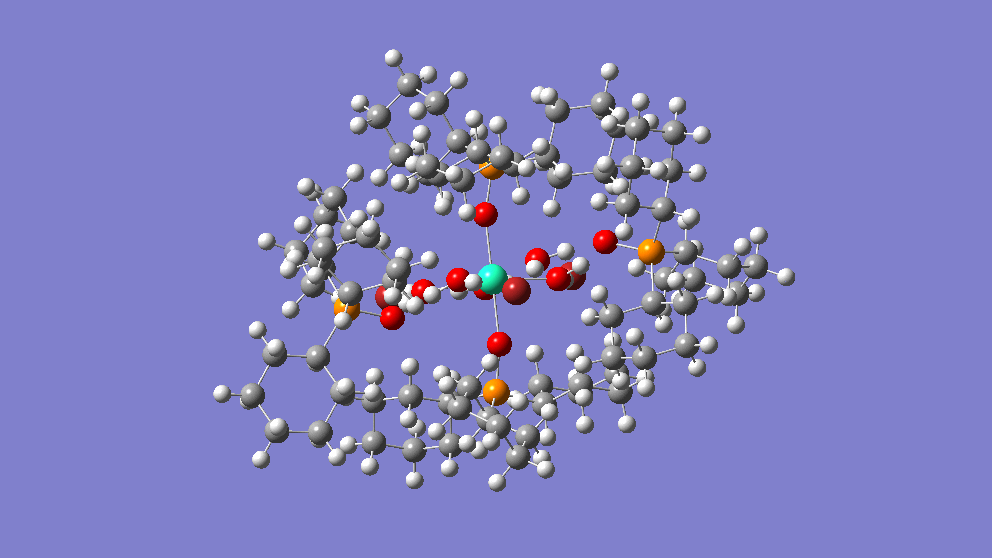

Supplement: Supplementary file 2 — Supplementary [file ANIE-59-8818-s002.zip › 20.gif]

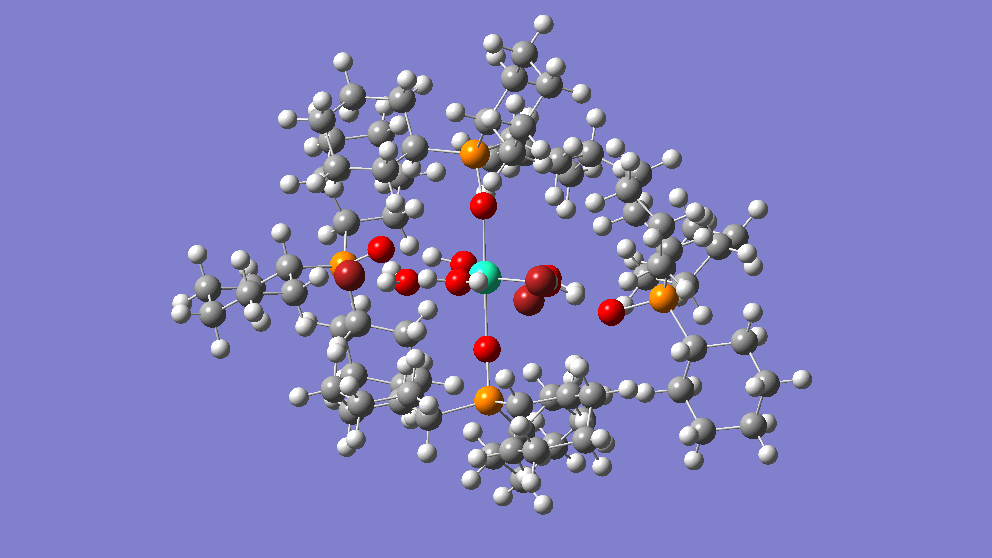

Supplement: Supplementary file 2 — Supplementary [file ANIE-59-8818-s002.zip › 200.gif]

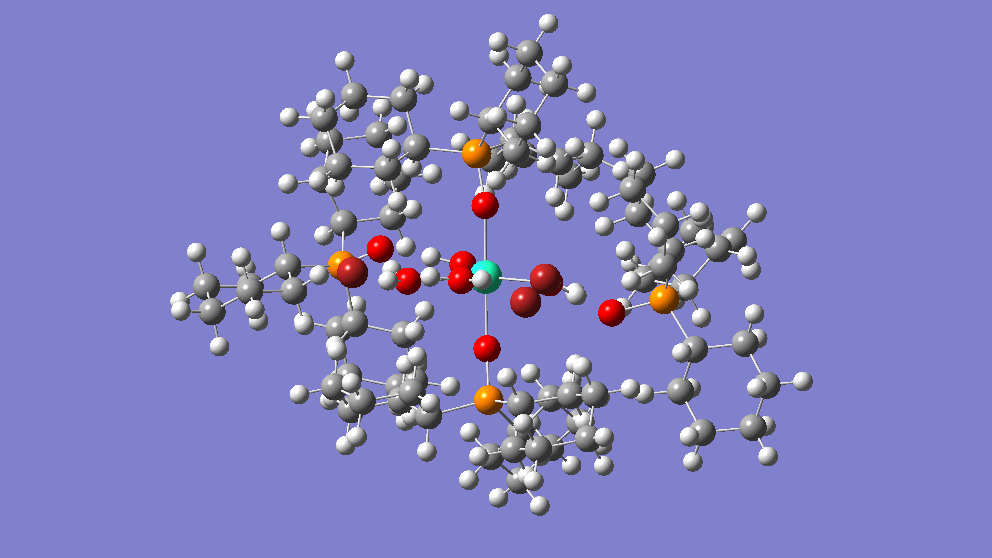

Supplement: Supplementary file 2 — Supplementary [file ANIE-59-8818-s002.zip › 209.gif]

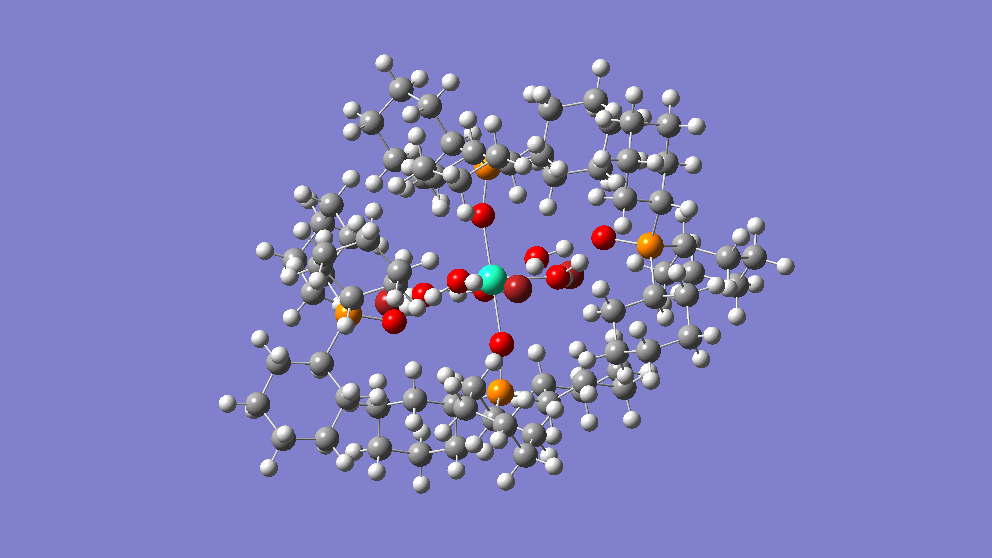

Supplement: Supplementary file 2 — Supplementary [file ANIE-59-8818-s002.zip › 22.gif]

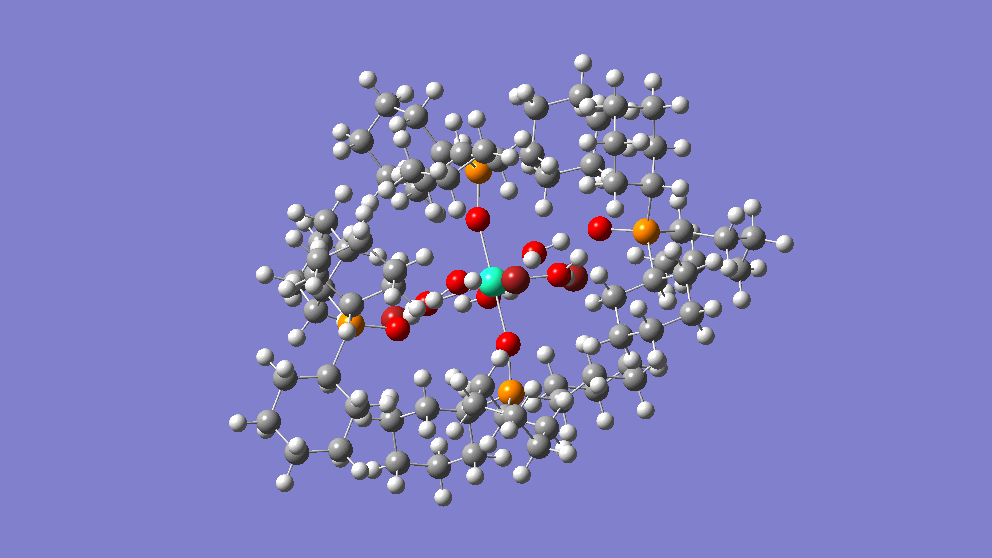

Supplement: Supplementary file 2 — Supplementary [file ANIE-59-8818-s002.zip › 23.gif]

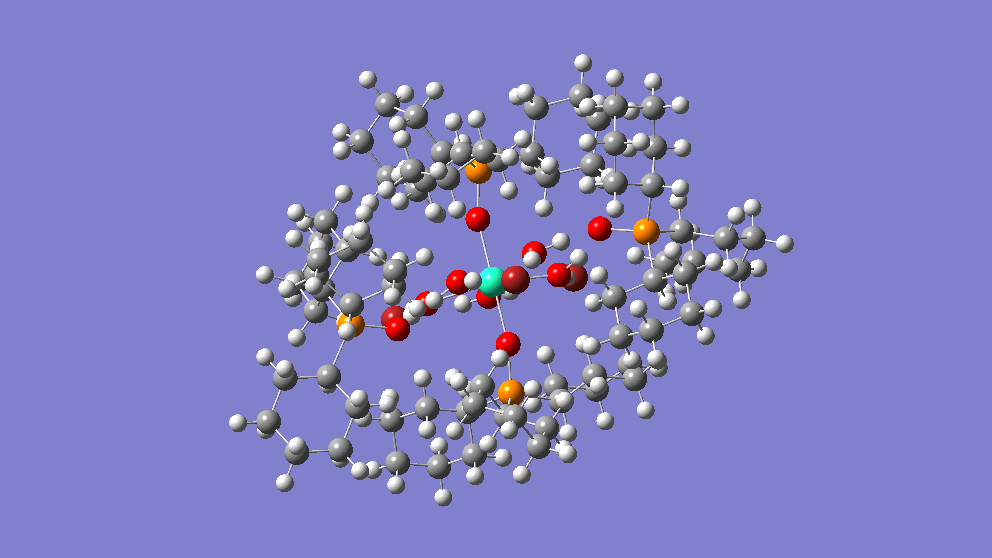

Supplement: Supplementary file 2 — Supplementary [file ANIE-59-8818-s002.zip › 25.gif]

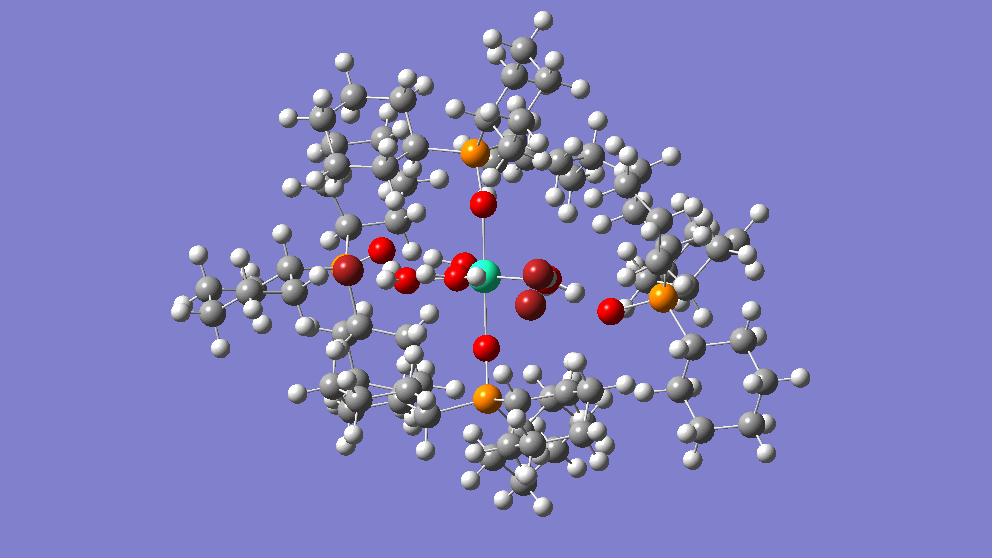

Supplement: Supplementary file 2 — Supplementary [file ANIE-59-8818-s002.zip › 308.gif]

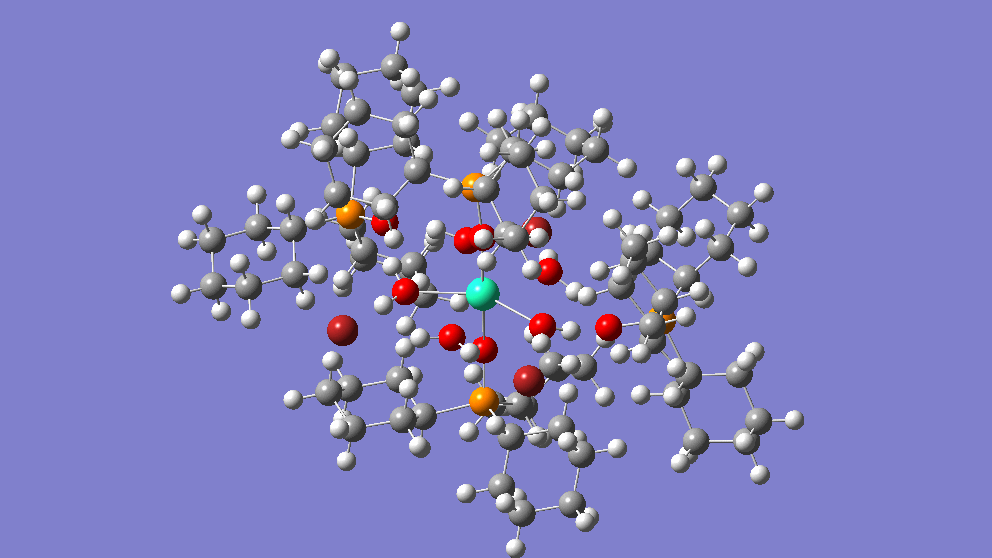

Supplement: Supplementary file 2 — Supplementary [file ANIE-59-8818-s002.zip › 313.gif]

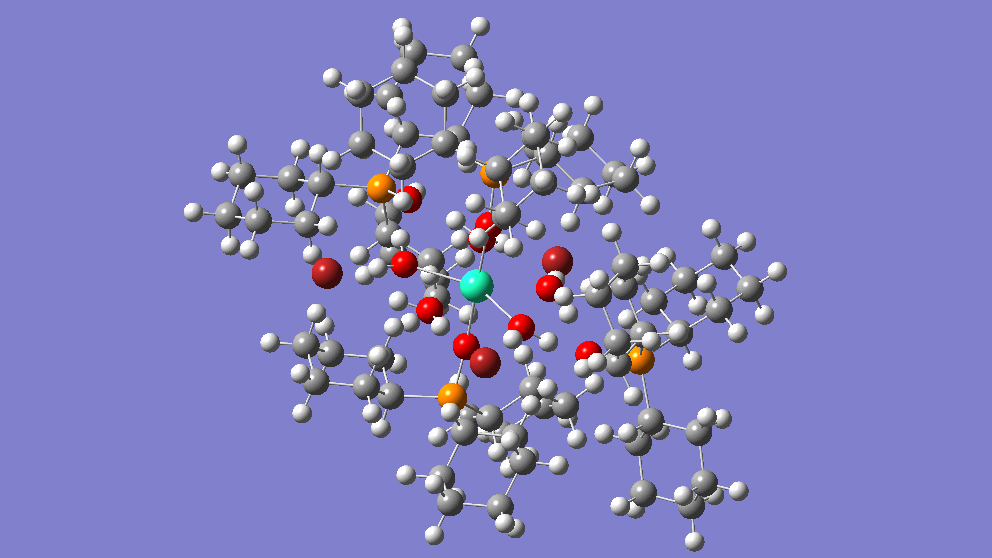

Supplement: Supplementary file 2 — Supplementary [file ANIE-59-8818-s002.zip › 373.gif]

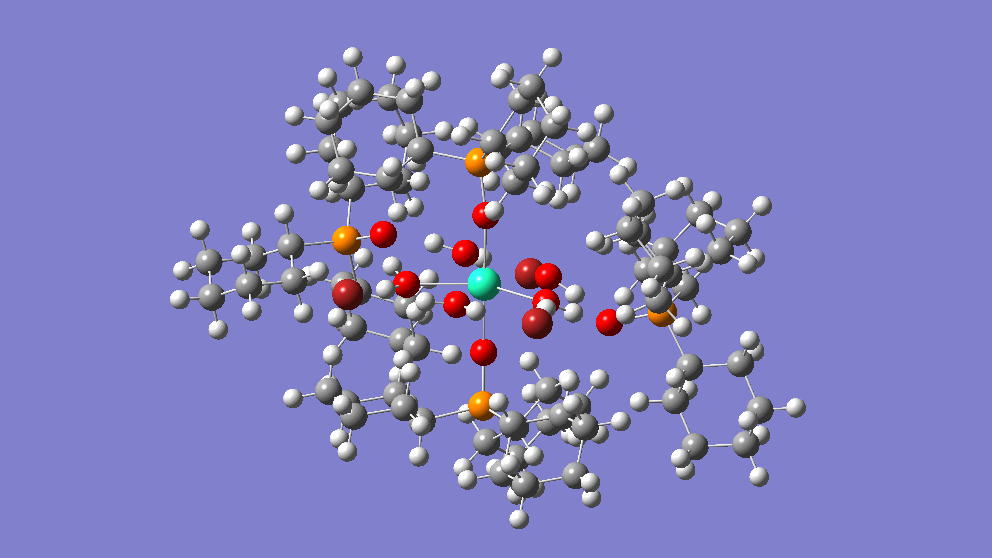

Supplement: Supplementary file 2 — Supplementary [file ANIE-59-8818-s002.zip › 375.gif]

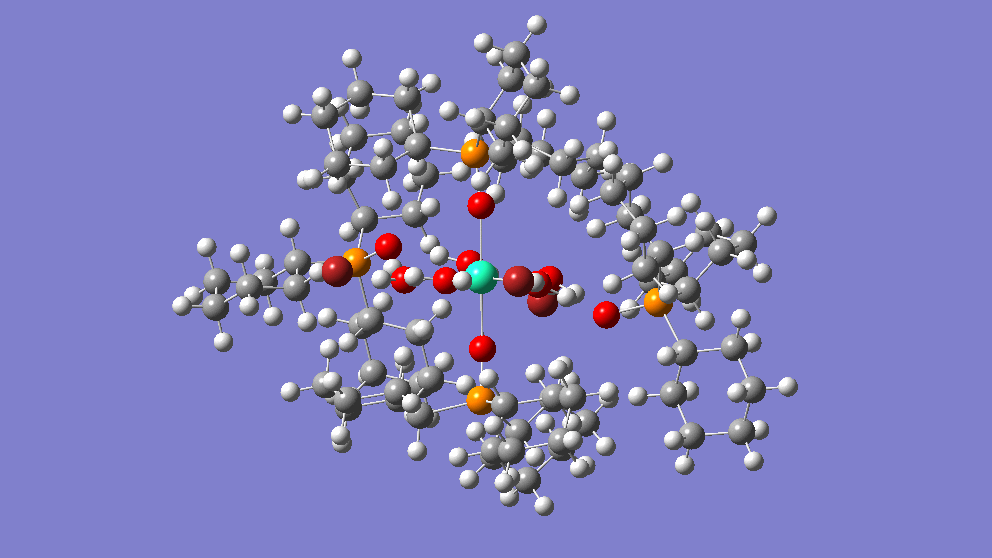

Supplement: Supplementary file 2 — Supplementary [file ANIE-59-8818-s002.zip › 386.gif]

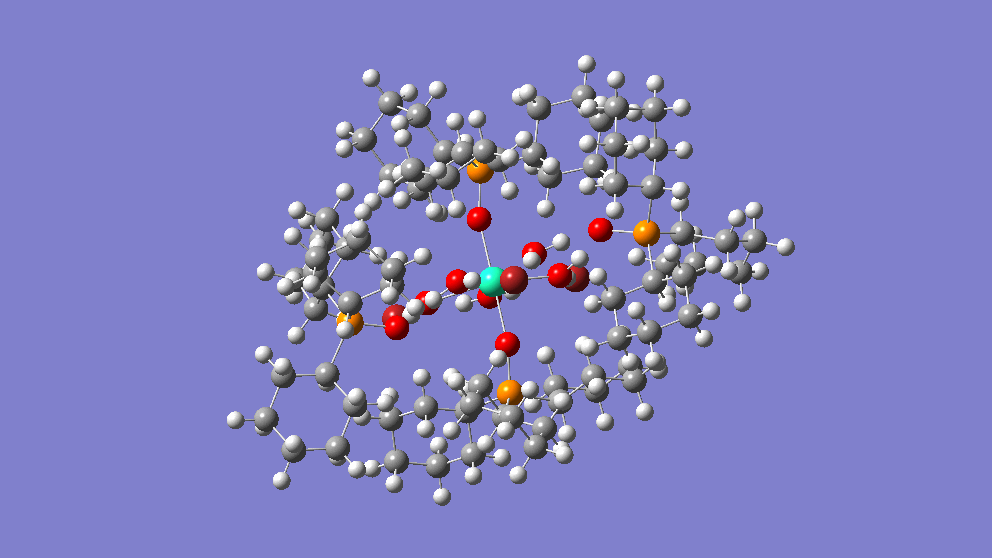

Supplement: Supplementary file 2 — Supplementary [file ANIE-59-8818-s002.zip › 43.gif]

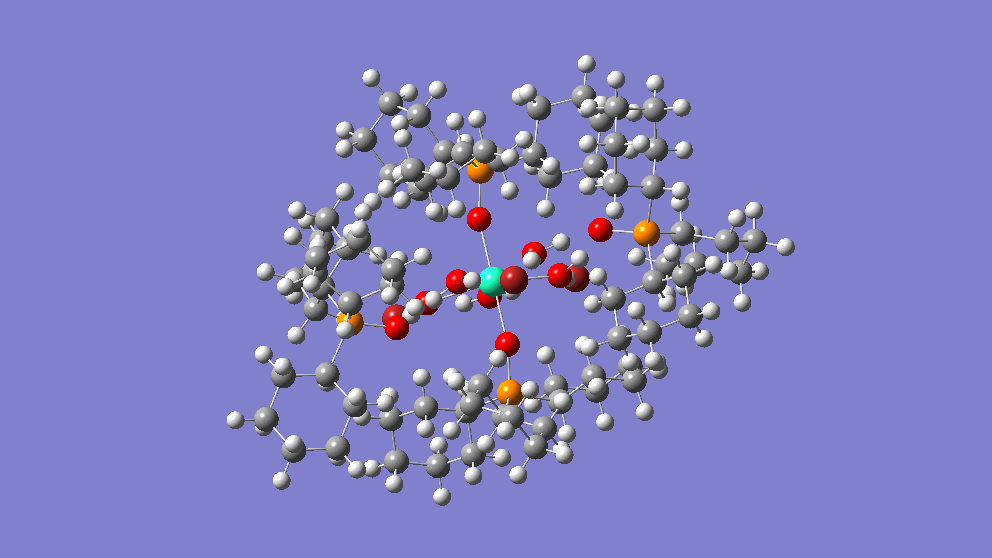

Supplement: Supplementary file 2 — Supplementary [file ANIE-59-8818-s002.zip › 45.gif]

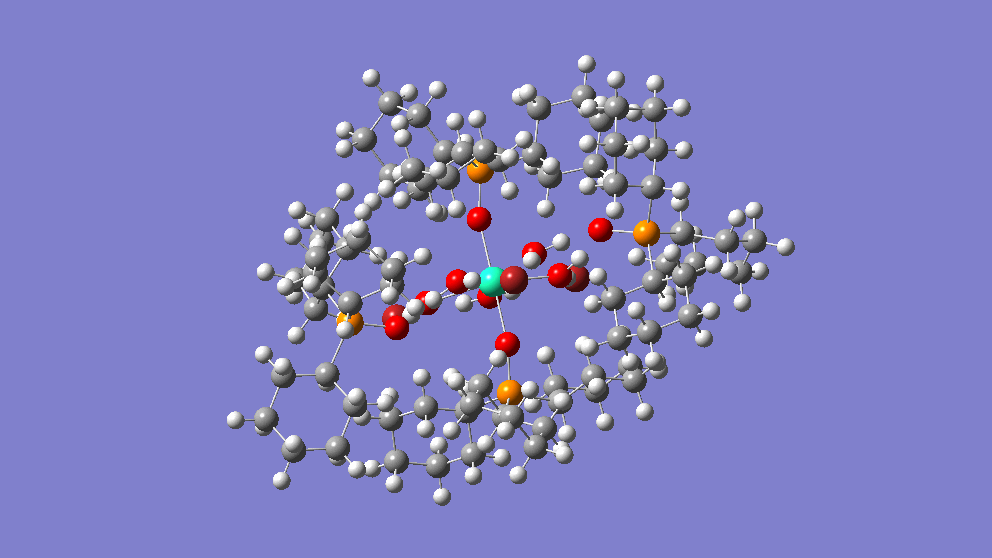

Supplement: Supplementary file 2 — Supplementary [file ANIE-59-8818-s002.zip › 47.gif]

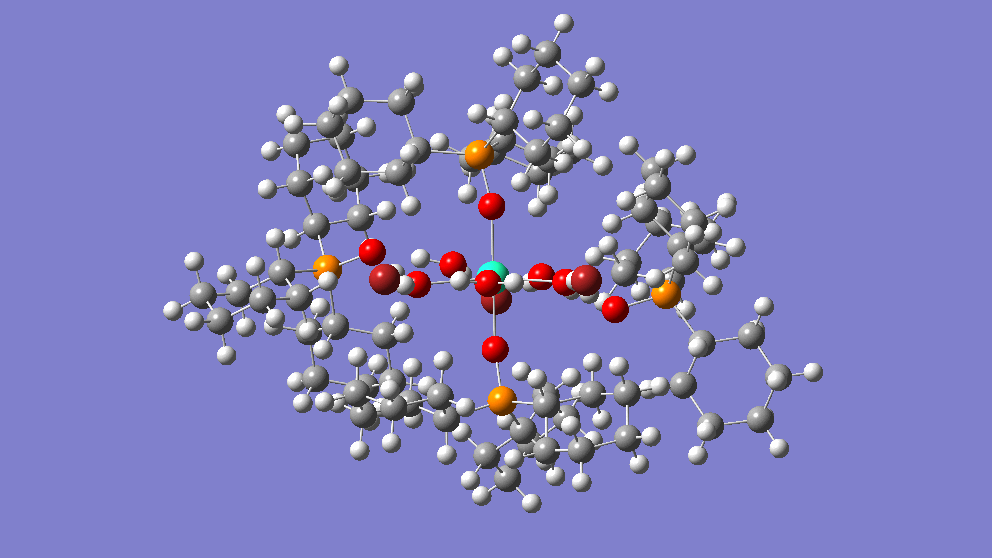

Supplement: Supplementary file 2 — Supplementary [file ANIE-59-8818-s002.zip › 60.gif]

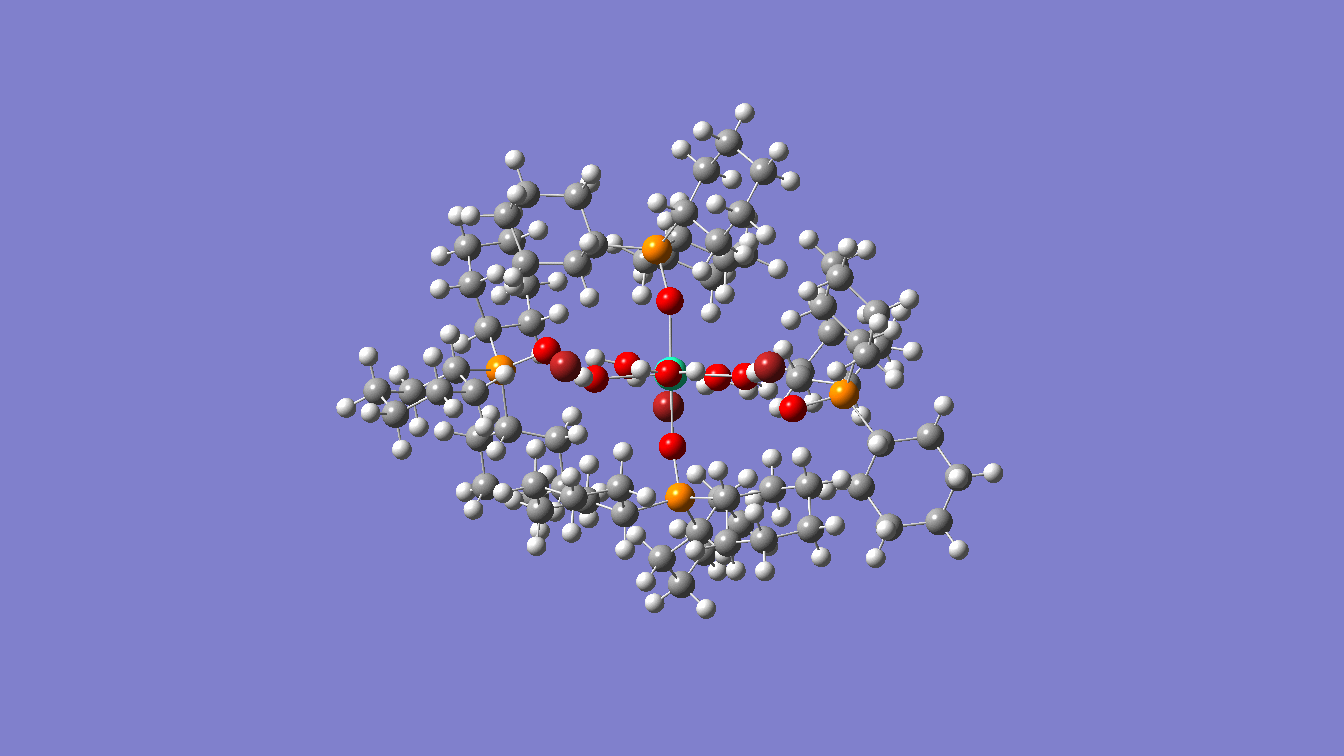

Supplement: Supplementary file 2 — Supplementary [file ANIE-59-8818-s002.zip › 61.gif]

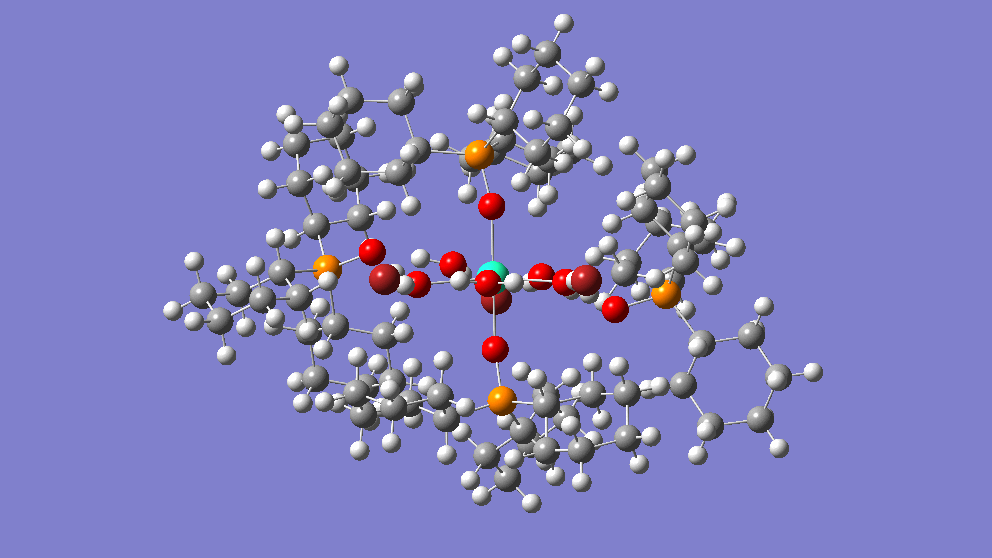

Supplement: Supplementary file 2 — Supplementary [file ANIE-59-8818-s002.zip › 62.gif]

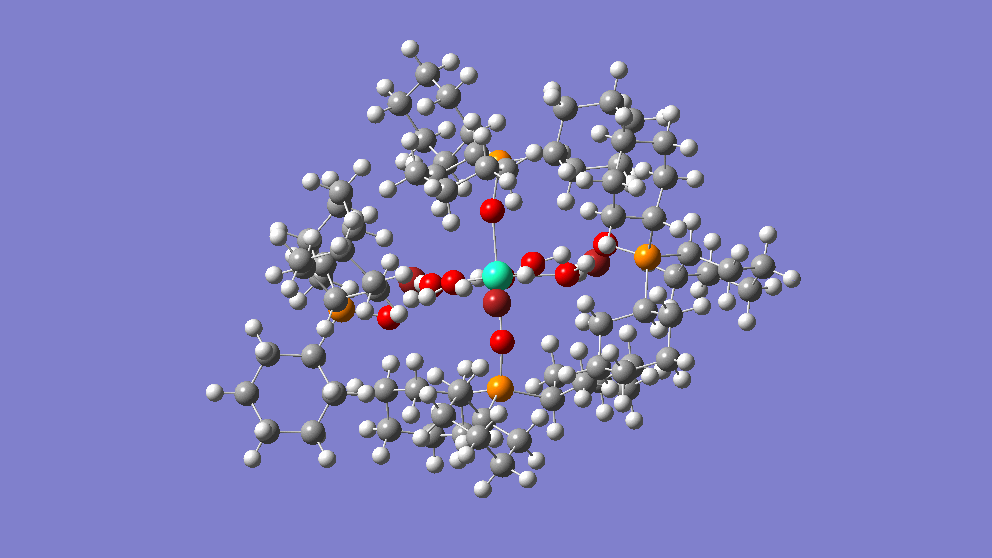

Supplement: Supplementary file 2 — Supplementary [file ANIE-59-8818-s002.zip › 7.gif]
